# Capsaicin ameliorate pulmonary fibrosis via antioxidant Nrf-2/ PPAR- γ pathway activation and inflammatory TGF-β1/ NF-κB/COX II pathway inhibition

**DOI:** 10.3389/fphar.2024.1333715

**Published:** 2024-02-21

**Authors:** Wesam H. Abdulaal, Hani Z. Asfour, Nawal Helmi, Hadeel Al Sadoun, Basmah Eldakhakhny, Nabil A. Alhakamy, Hani Mohammed Alqarni, Saeed Ali Mohammed Alzahrani, Mohamed A. El-Moselhy, Sara S. Sharkawi, Esam Mohamed Aboubakr

**Affiliations:** ^1^ Department of Biochemistry, King Fahd Medical Research Center, Faculty of Science, Cancer and Mutagenesis Unit, King Abdulaziz University, Jeddah, Saudi Arabia; ^2^ Mohamed Saeed Tamer Chair for Pharmaceutical Industries, Faculty of Pharmacy, King Abdulaziz University, Jeddah, Saudi Arabia; ^3^ Center of Excellence for Drug Research and Pharmaceutical Industries, King Abdulaziz University, Jeddah, Saudi Arabia; ^4^ Department of Medical Microbiology and Parasitology, Faculty of Medicine, King Abdulaziz University, Jeddah, Saudi Arabia; ^5^ Department of Biochemistry, College of Science, University of Jeddah, Jeddah, Saudi Arabia; ^6^ Department of Medical Laboratory Sciences, Faculty of Applied Medical Sciences, King Abdulaziz University, Jeddah, Saudi Arabia; ^7^ Department of Clinical Biochemistry, Faculty of Medicine, King Abdulaziz University, Jeddah, Saudi Arabia; ^8^ Department of Pharmaceutics, Faculty of Pharmacy, King Abdulaziz University, Jeddah, Saudi Arabia; ^9^ Clinical Pharmacy and Pharmacology Department, Ibn Sina National College for Medical Studies, Jeddah, Saudi Arabia; ^10^ Department of Pharmacology and Toxicology, Faculty of Pharmacy, Minia University, Minia, Egypt; ^11^ Department of Pharmacology and Toxicology, Faculty of Pharmacy, South Valley University, Qena, Egypt

**Keywords:** Capsaicin, bleomycin, Pirfenidone, pulmonary fibrosis, TGF-β1, PPAR-γ, TNF-α

## Abstract

Bleomycin is an effective antibiotic with a significant anticancer properties, but its use is limited due to its potential to induce dose-dependent pulmonary fibrosis. Therefore, this study aimed to assess the therapeutic potential of Capsaicin as an additional treatment to enhance patient tolerance to Bleomycin compared to the antifibrotic drug Pirfenidone. Pulmonary fibrosis was induced in rats through by a single intratracheal Bleomycin administration in day zero, followed by either Capsaicin or Pirfenidone treatment for 7 days. After the animals were sacrificed, their lungs were dissected and examined using various stains for macroscopic and histopathological evaluation. Additionally, the study assessed various antioxidant, anti-inflammatory, and antifibrotic parameters were assessed. Rats exposed to Bleomycin exhibited visible signs of fibrosis, histopathological alterations, increased collagen deposition, and elevated mucin content. Bleomycin also led to heightened increased inflammatory cells infiltration in the bronchoalveolar lavage, elevated fibrosis biomarkers such as hydroxyproline, alpha-smooth muscle actin (α-SMA) and transforming growth factor-beta (TGF-β1), increased inflammatory markers including tumor necrosis factor-alpha (TNF-α), interlukine-6 (Il-6), interlukine-1β (Il-1β) nuclear factor-kappa B (NF-κB), and Cyclooxygenase-2 (COX-2), and transforming growth factor-beta (TGF-β1),. Furthermore, it reduced the expression of peroxisome proliferator-activated receptor-gamma (PPAR-γ), increased oxidative stress biomarkers like nitric oxide (NO), malondialdehyde (MDA), myeloperoxidase (MPO) and protein carbonyl. Bleomycin also decreased the expression of nuclear factor erythroid 2–related factor 2 (Nrf-2), reduced glutathione (GSH), total antioxidant capacity, and the activities of catalase and superoxide dismutase (SOD). Treating the animals with Capsaicin and Pirfenidone following Bleomycin exposure resulted in improved lung macroscopic and microscopic characteristics, reduced collagen deposition (collagen I and collagen III) and mucin content, decreased inflammatory cell infiltration, lowered levels of hydroxyproline, α-SMA, and TGF-β1, decreased TNF-α, Il-6, Il-1β, NF-κB, and COX-2, increased PPAR-γ and Nrf-2 expression, and improvement improved in all oxidative stress biomarkers. In summary, Capsaicin demonstrates significant antifibrotic activity against Bleomycin-induced lung injury that may be attributed, at least in part, to the antioxidant and anti-inflammatory activities of Capsaicin mediated by upregulation of PPAR-γ and Nrf-2 expression and decreasing. TGF-β1, NF-κB and COX II proteins concentrations.

## 1 Introduction

Bleomycin is a highly effective chemotherapy used extensively in the treatment of different types of cancer including; Hodgkin lymphoma and testicular germ-cell tumors. However, bleomycin has the potential to cause serious and life-threatening damage to the lungs (Ayilya et al., 2023). This can manifest as several conditions, including hypersensitivity pneumonitis, bronchiolitis obliterans organizing pneumonia (BOOP), acute interstitial pneumonia, and progressive pulmonary fibrosis (Demirkol et al., 2023). Pulmonary toxicity is a recognized adverse effect of anticancers, nevertheless, a mortality rate of 1%–4% from bleomycin is considered unacceptable for patients with treatable malignancies. The toxic effects of bleomycin are primarily caused by the generation of free radical and its lung specificity caused by bleomycin catalyzing hydrolase, which was not found in lung tissue, rendering this organ vulnerable to bleomycin toxicity (Usman et al., 2010; Ishida et al., 2023).

The development of lung fibrosis is mostly driven by acute inflammation; including significant upregulation of mononuclear macrophages, lymphocytes, and neutrophils. Bleomycin induces the release of inflammatory cytokines, TNF-α, IL-1, IL-18, IL-22, and IL-17a, from alveolar macrophages (Kadam and Schnitzer, 2023). It also stimulates endothelial cells to secrete IL-6. Cytokines stimulate lymphocytes and increase the expression of adhesion molecules on endothelial cells, which allows inflammatory cells to stick to the endothelium, enter the interstitium, and damage endothelial cells via the Fas-FasL pathway (Yoshizaki et al., 2010; Altintas et al., 2016). The activation of fibroblasts in pulmonary fibrosis occurs at early stage due to the stimulation of fibronectin. This stimulation might be caused by injured endothelial cells or by the presence of cytokines such as TNF-α, platelet derived growth factor (PDGF), and transforming growth factor-β (TGF-β) (Kabel et al., 2016; Chen et al., 2023). Prolonged exposure of the lungs to bleomycin can result in elevated production of collagen and the accumulation of different matrix proteins such as collagens, elastin, and proteoglycans. In addition, alveolar macrophages that have been stimulated by bleomycin promote the production of hyaluronan (Venkatesan et al., 2011; Wang et al., 2023). T cells also contribute to the lung damage caused by inflammation. Cytokines, such as IFN-γ and IL-13, are released during Th1 and Th2 inflammation, respectively (Lei et al., 2016).

Capsaicin, also known as trans-8-methyl-N-vanillyl-6-nonenamide, is the primary and most potent alkaloid in the capsaicinoid group, responsible for the characteristic chili flavor found in chili peppers (Ilie et al., 2019). Capsaicin offers substantial health benefits, including analgesic, anticancer, neuroprotective, and gastroprotective properties, which can largely be attributed to its antioxidant and anti-inflammatory characteristics. Furthermore, Capsaicin has been shown to modulate macrophage function and reduce the release of proinflammatory cytokines, reactive oxygen species (ROS), proteases, and lysosomal enzymes (Zimmer et al., 2012). Additionally, several studies highlighted the antifibrotic effect of Capsaicin through the inhibition of TGF-β1 signaling in various models of fibrosis such as kidney fibrosis (Liu et al., 2022).

Therefore, the present study aims to investigate the potential therapeutic effects of Capsaicin in mitigating Bleomycin-induced pulmonary toxicity in rats and elucidating the underlying mechanisms involved.

## 2 Materials and methods

### 2.1 Drugs and chemicals

Capsaicin, Bleomycin sulfate and Pirfenidone were procured from Sigma Aldrich (St. Louis, MO, United States) under the catalog numbers M2028, BP971, and P2116, respectively. All chemicals utilized in this study were of the highest quality available in the commercial market.

### 2.2 Animals

A total of 32 adult male Sprague Dawley rats, aged 10–12 weeks and weighing between 140 and 160 g, were sourced from the National Research Centre (NRC) in Giza, Egypt, for inclusion in this study. To allow the rats to adapt to the laboratory environment, they were acclimated for 1 week before the commencement of the experiment. Throughout the study, the animals were maintained under controlled conditions, with a temperature of 28 ± 2°C, humidity 55 ± 5% and a 12-h light/dark cycle. They were provided with standard chow containing 20% protein, 8% fiber, 4% fat, 7% ash, and approximately 60% carbohydrates, along with *ad libitum* access to water. All procedures involving animals were carried out following approval from the ethical committee of the Faculty of Pharmacy at South Valley University, Egypt, under approval number (P.S.V.U 112/23), in accordance with international guidelines for the use of experimental animals (Animal Research: Reporting of *In vivo* Experiments) guidelines.

### 2.3 Experimental design

The animals were randomly divided into four groups, each consisting of 8 rats. They were assigned to different treatment groups: 1) Group one, serving as the control, received only the vehicle solutions for Capsaicin and Bleomycin. 2) Group two, the Bleomycin-only-treated group, received Bleomycin (5 U/kg body weight) (Razzaque et al., 1998) along with the vehicle solutions for Capsaicin and Pirfenidone (0.5% sodium carboxymethyl cellulose, CMC). 3) Group three received Bleomycin and Pirfenidone (50 mg/kg, administered orally) (Guo et al., 2019). 4) Group four received Bleomycin and Capsaicin (3 mg/kg, administered orally) (Lee et al., 2003). Anesthesia was administered to the animals, following which a single dose of Bleomycin (5 mg/kg) dissolved in saline was introduced via intratracheal instillation on day zero to induce pulmonary fibrosis. Conversely, the control rats were administered equivalent volumes of saline via the same route. Capsaicin and Pirfenidone were administered orally to the animals starting from day one and continued for 7 days. Upon concluding the experiment, the rats were anesthetized using thiopental sodium to collect bronchoalveolar lavage fluid. Subsequently, blood samples were collected from the retro-orbital venous plexus. These blood samples were maintained at room temperature until serum separation, accomplished by centrifugation at 3,000 rpm for 15 min. The sera were then collected and stored at −80°C for subsequent biochemical analyses. The lungs were removed, rinsed with ice-cold saline, and weighed to determine the lung/body weight index for macroscopic examination. The left lobe of each animal’s lung was used to prepare lung tissue homogenate, while the right lobe was isolated for histopathological examination.

### 2.4 Collection of bronchoalveolar lavage fluid (BALF)

Under deep anesthesia, the thoracic cage was opened, revealing the rat tracheas, which were then exposed and cannulated. The lungs were subsequently flushed with 6 mL of 0.9% saline administered in 2 mL increments. Gentle chest compressions were performed to recover the saline. The bronchoalveolar lavage fluid (BALF) was collected and centrifugated at 2,000 rpm, maintained at 4°C for 10 min. The cells that settled were then resuspended in 500 μL of saline to assess the levels of inflammatory cells, including total and differential cell counts, following the method described by Henderson in 2005 (Henderson and Pathology, 2005).

### 2.5 Histopathological examination

The lung tissues of the rats were initially fixed in a 10% formaldehyde solution for 48 h. Subsequently, they underwent a dehydration process involving successive ethanol dilutions before being embedded in paraffin. The paraffin-embedded lung tissues were then sectioned into slices of 5 μm thickness using a sliding microtome. These tissue sections were subjected to staining procedures, including hematoxylin and Eosin (H and E), Masson trichrome stains (MTC), and periodic acid Schiff (PAS) stains, for histopathological examination. Examination of the slides was carried out employing a light microscope manufactured by Olympus Opticals in Tokyo, Japan, at a magnification of ×200. The degree of pulmonary fibrosis was assessed based on the MTC-stained tissue sections using the following grading system: Grade 0: Indicates normal lung tissue. Grade 1: Signifies minimal fibrous changes in alveoli or bronchial walls. Grade 2: Reflects moderate thickening of alveoli or bronchial walls without disruption of lung architecture. Grade 3: Represents tissue fibrosis accompanied by damage to lung architecture. Grade 4: Denotes severe distortion and architectural damage in addition to forming fibrous areas. Grade 5: Corresponds to total lung fibrosis. The fibrosis score was determined by analyzing ten fields within the lung sections. The quantitative evaluation of mucus production was expressed as the area filled with mucus relative to the total airway lumen area in the PAS-stained tissue sections.

### 2.6 The assessment of hydroxyproline content

The quantification of hydroxyproline content in lung homogenates was conducted by utilizing a kit acquired from Sigma Aldrich (St. Louis, MO, United States, Cat # MAK008), following the guidelines provided by the manufacturer. This assay relies on the oxidation of free hydroxyproline using chloramine-T, leading to the creation of a pyrrole compound that generates a chromophore when Ehrlich’s reagent is introduced. The resulting product’s absorbance can then be measured at 550 nm, as previously described (Cissell et al., 2017).

### 2.7 Colorimetric assessments of the oxidative stress biomarkers

We assessed various parameters, including lipid peroxidation, which was quantified using malondialdehyde (MDA), a major thiobarbituric acid reactive species. Additionally, we assessed carbonyl content, reduced glutathione (GSH), total antioxidant capacities (TAC), Myeloperoxidase (MPO) as well as catalase and superoxide dismutase (SOD) activities. These assessments were carried out in accordance with the manufacturer’s instructions, employing commercially available kits as follows: MDA assay kit (Biodiagnostics, Egypt, Cat # MD2529), Protein Carbonyl Colorimetric Assay Kit (Cayman, Ann Arbor, Michigan, United States, Cat # 10005020), GSH assay kit (Biodiagnostics, Egypt, Cat # GR2511), TAC assay kit (Biodiagnostics, Egypt, Cat # TA2513), Myeloperoxidase (Abcam, Cambridge, United Kingdom, Cat # ab105136), Catalase activity assay kit (Biodiagnostics, Egypt, Cat # SD2521), and SOD activity assay kit (Biodiagnostics, Egypt, Cat # CA2517).

### 2.8 The assessment of the nitrite content

We measured nitrite content, which is indicative of nitric oxide (NO), through a colorimetric method using the commercially available NO assay kit (Biodiagnostics, Egypt, Cat # NO2533). This assay relies on converting NO into nitrous acid, which subsequently reacts with sulfanilamide and N-(1-naphthyl) ethylenediamine to produce an azo dye. The absorbances of the samples were then determined colorimetrically at a wavelength of 540 nm (Archer, 1993).

### 2.9 Assessment of inflammatory biomarker

We quantified the lung tissue protein content of tumor necrosis factor-alpha (TNF-α) employing a solid-phase sandwich ELISA method. This assay utilized the Rat Tumor Necrosis Factor-α ELISA Kit (Sigma Aldrich, United States, Cat # RAB0480) and followed the manufacturer’s instructions. Moreover, the level of the inflammatory mediators TNF- α, (Sigma Aldrich, United States, Cat # RAB0480), IL-1β (Sigma Aldrich, United States, Cat # RAB0273) and IL-6 (Sigma Aldrich, United States, Cat # RAB0311) were determined in the BALF, following the manufacturer instructions.

### 2.10 Immunohistochemical staining of α-SMA, Nrf-2, NF-Kβ, COX 2, TGF-β1, and PPAR-γ in the rats’ lung tissue

The expression of various proteins, including alpha-smooth muscle actin (α-SMA), nuclear factor erythroid 2–related factor 2 (Nrf-2), nuclear factor-kappa B (NF-Kβ), cyclooxygenase-2 (COX-2), transforming growth factor-beta 1 (TGF-β1), and Peroxisome proliferator-activated receptor gamma (PPAR-γ), in the lung tissues was assessed through immunohistochemical staining. To perform this analysis, sections of lung tissue obtained from paraffin blocks were sliced into 5 μm thick sections. These sections then underwent deparaffinization and antigen retrieval by heating in steaming water at 80°C for 20 min. Washing steps, using 0.1 M PBS at pH 7.4, were carried out for 5 min between each stage of the process. Subsequently, the lung sections were incubated for 18 h with appropriately diluted rabbit polyclonal primary antibodies, namely, anti-α-SMA, anti-Nrf-2, anti-NF-Kβ, anti-COX 2, anti-TGF β1, and anti-PPAR-gamma (obtained from Abcam, Cambridge, United Kingdom, Cat # ab220164, ab31163, ab76302, ab179800, ab215715, and ab310323, respectively). Following incubation, the slides were washed and then incubated with a secondary antibody, HRP Anti-Rabbit IgG antibody (Abcam, Cambridge, United Kingdom, Cat # ab288151). To stain the target antigen, the tissue staining anti-rabbit kit (Abcam, Cambridge, United Kingdom, Cat # ab64261) was employed. Visual examination of the immune-stained lung tissues was conducted using a light microscope (Olympus Opticals, Tokyo, Japan). A total of five fields per slide were analyzed, scored, and quantified using Image-Pro Plus 5.0 image analysis software from the National Institutes of Health, located in Bethesda, MD, United States.

### 2.11 Western blot analysis

Tissue proteins were extracted using RIPA buffer (Beyotime) and their quantities were determined. Equal quantities of proteins were separated on 10% SDS-PAGE gel, proteins were transferred onto PVDF membranes (EMD Millipore, Billerica, MA, United States), treated with 5% BSA for 1 h at room temperature to stop further reactions. The membranes were subsequently placed at 4°C and treated overnight with primary antibodies; α-SMA (ab5694, Abcam), collagen I (ab260043, Abcam), and collagen III (ab7778, Abcam), membranes were washed using TBST solution and incubated with HRP-conjugated secondary antibodies for duration of 1 h at 36°C. The bands’ intensity was determined by chemiluminescence technique, the obtained images were analyzed using ImageJ program.

### 2.12 Statistical analyses

For parametric data, we expressed the results as the mean ± standard deviation (SD), and we determined statistical significance using One-way ANOVA followed by Tukey’s multiple comparison test. Nonparametric data were presented as the median along with the interquartile range, and we analyzed them using the Kruskal–Wallis test, followed by Dunn’s *post hoc* test. The data analyses were conducted using GraphPad Prism software (Prism 8.1, GraphPad Software), with statistical significance defined as *p* < 0.05.

## 3 Results

### 3.1 The effects of Bleomycin, Pirfenidone, and Capsaicin on the white blood cell count in bronchoalveolar lavage fluid of the treated rats

Bleomycin administration resulted in a notable increase in both the total cell count and the proportions of macrophages, neutrophils, eosinophils, and lymphocytes in the bronchoalveolar lavage fluid (BALF) of the treated rats when compared to the control group. Conversely, treatment with Pirfenidone significantly reduced the total cell count and the proportions of neutrophils and eosinophils compared to the Bleomycin-only-treated rats. Of particular interest, Capsaicin treatment elicited a significant decrease in the total cell count and in the proportions of macrophages, neutrophils, and eosinophils compared to the Bleomycin-only-treated rats. Remarkably, these values in the Capsaicin-treated group nearly approached those of the control group, as illustrated in Table 1.

**TABLE 1:** The effect of Bleomycin, Pirfenidone, and Capsaicin on the white blood cell count in bronchoalveolar lavage fluid.

Groups	Total cells (×106/mL)	Macrophage (%)	Neutrophils (%)	Eosinophil (%)	Lymphocytes (%)
Control	0.49 ± 0.06	89.2 ± 7.6	4.1 ± 0.55	2.2 ± 0.3	4.1 ± 0.32
Bleomycin	1.3* ± 0.12	62.3* ± 5.5	28.1* ± 2.3	3.3* ± 0.41	8.8* ± 0.7
Bleomycin + Pirfenidone	1.1^#^ ± 0.13	68.4 ± 7.1	25.6^#^ ± 2.1	2.8^#^ ± 0.19	9.68 ± 0.8
Bleomycin + Capsaicin	0.65^#,$^ ± 0.04	81.4^#,$^ ± 9.9	6.2^#,$^± 0.3	1.6^#,$^± 0.11	8.9 ± 1.15

Bleomycin (5 mg/kg) was administered via intratracheal instillation on day zero. Capsaicin (3 mg/kg) and Pirfenidone (50 mg/kg) were orally administered daily for 7 days. Results were analyzed by one-way ANOVA followed by Tukey’s as a *post hoc* test (*n* = 8). *, #, and $ are considered statistically significant from the control, Bleomycin-only, and Bleomycin-plus Pirfenidone-treated group, respectively, at *p < 0.0*5.

### 3.2 The effects of Bleomycin, Pirfenidone, and Capsaicin on the macroscopic features of the lungs of the treated rats

**FIGURE 1:**
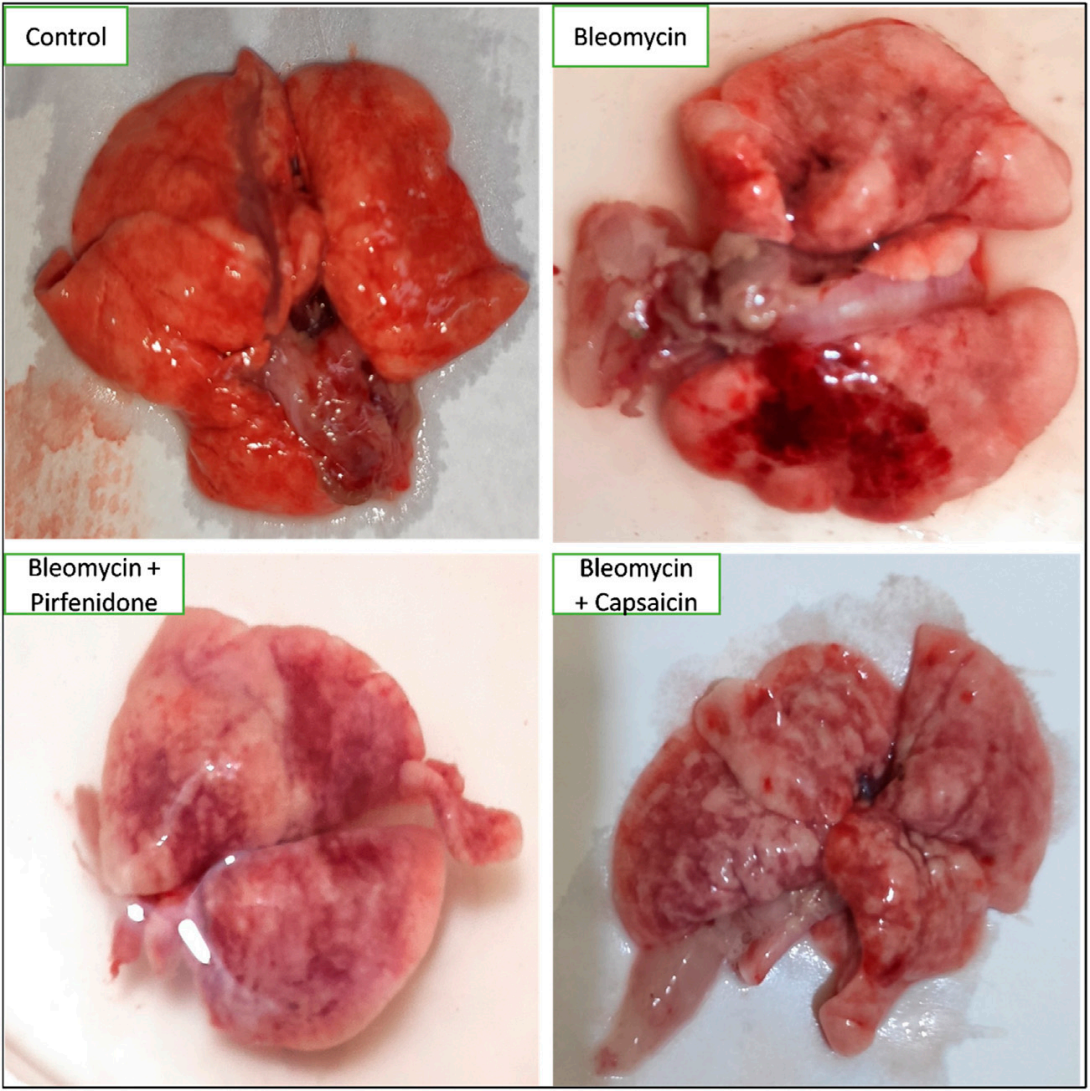
The effects of Bleomycin, Capsaicin, and Pirfenidone on the morphology of the rat lungs. Bleomycin (5 mg/kg) was administered via intratracheal instillation on day zero. Capsaicin (3 mg/kg) and Pirfenidone (50 mg/kg) were orally administered daily for 7 days.

In the control group, the lungs displayed a typical, healthy morphology characterized by a smooth and uniform surface. In contrast, the Bleomycin-treated group exhibited irregularities, coarseness, interstitial hemorrhagic lesions, atrophic alterations, tissue degeneration, notable tissue congestion, collapsed lung areas, and tissue consolidation, resulting in a loss of tissue elasticity. Treatment with Pirfenidone and Capsaicin was observed to enhance the macroscopic appearance of the lungs, leading to improved surface smoothness and elasticity. Additionally, there were decreased levels of congestion, hemorrhagic lesions, and tissue degeneration, as depicted in Figure 1.

### 3.3 The effects of Bleomycin, Pirfenidone, and Capsaicin on the histopathological features of the lung tissues of the treated rats

**FIGURE 2:**
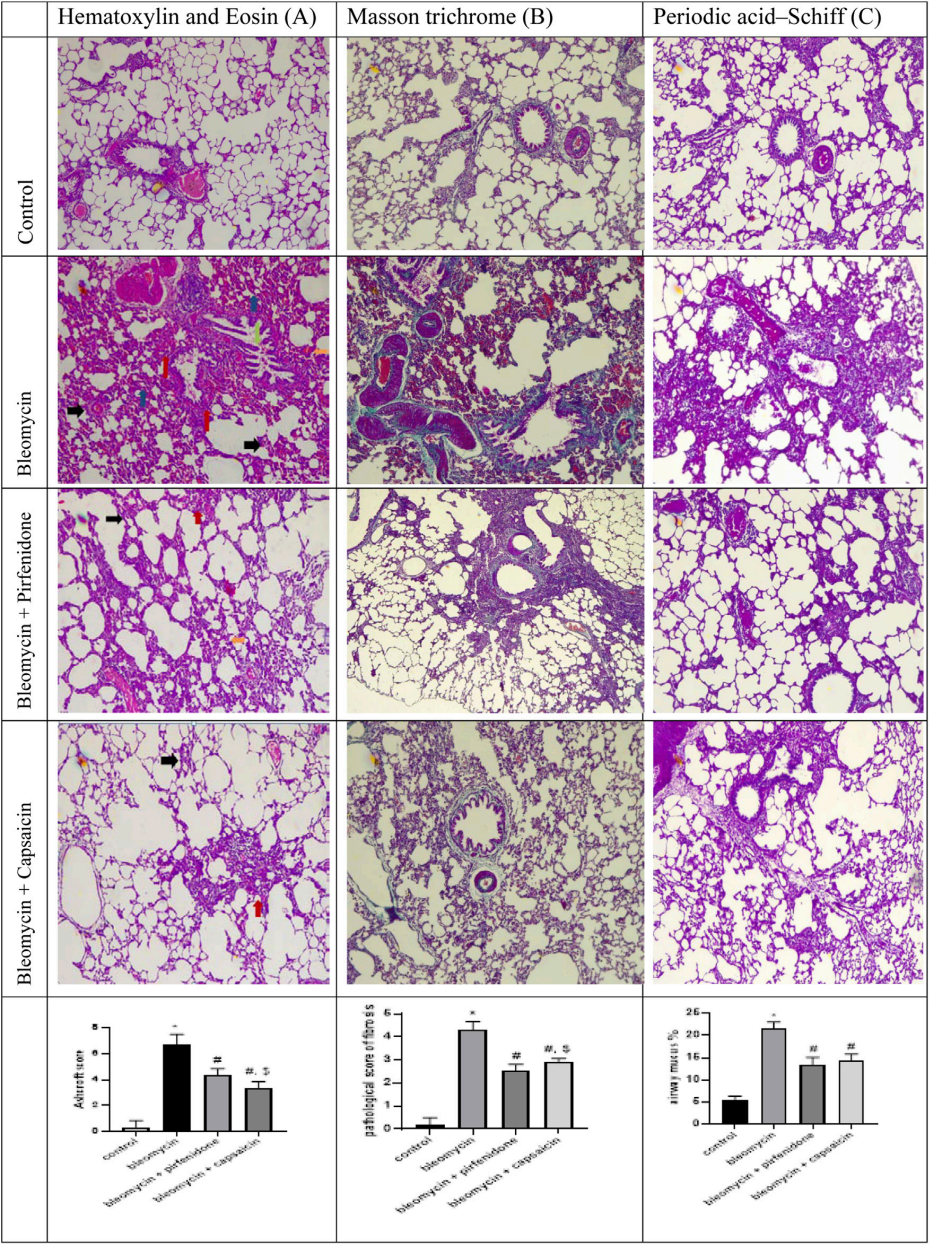
The effects of Bleomycin, Capsaicin, and Pirfenidone on the histopathological features of the rat lungs examined by hematoxylin and Eosin stain (Black arrow indicates degenerative alterations, blue arrows indicate predominantly collapsed alveoli, orange arrow indicates thickened inter-alveolar septa, red arrow indicates congested pulmonary blood vessels and green arrow indicates extensive cellular infiltration) **(A)**, Masson trichrome **(B)**, and Periodic acid–Schiff **(C)**. Bleomycin (5 mg/kg) was administered via intratracheal instillation on day zero. Capsaicin (3 mg/kg) and Pirfenidone (50 mg/kg) were administered orally for 7 days. Results were analyzed by one-way ANOVA followed by Tukey’s as a *post hoc* test (*n* = 8). *, #, and $ are considered statistically significant from the control, Bleomycin-only, and Bleomycin-plus Pirfenidone-treated group, respectively, at *p* < 0.05.

Staining with H and E (Figure 2A) highlighted that lung tissues from the control group displayed a normal architecture characterized by thin inter-alveolar septa, patent alveoli, healthy pneumocytes of types I and II, normal nuclei, and unobstructed bronchioles lined with regular columnar epithelium. In contrast, the Bleomycin-treated group exhibited extensive tissue damage, degenerative alterations, and distortion of pulmonary structure. Alveoli were predominantly collapsed, inter-alveolar septa thickened significantly, and there was extensive cellular infiltration and blood congestion. The bronchioles displayed severe infiltration of lymphocytic cells, with noticeable shedding of bronchiolar epithelial cells into the lumen. The Pirfenidone-treated group demonstrated moderate lung tissue damage, notably congested pulmonary blood vessels, mild thickening of intra-alveolar septa, and profound infiltration of inflammatory cells. However, in the lung tissue of Capsaicin-treated rats, the degree of destruction was less pronounced than in the Bleomycin-only and Bleomycin-plus-Pirfenidone-treated groups.

Furthermore, mild inflammatory cell infiltration was observed. Blood vessels were not congested, and bronchiolar epithelium shedding was moderate. In some areas of lung tissue, inter-alveolar septa were slightly thickened. Subsequent staining with MTC to assess collagen deposition revealed that the control group exhibited minimal collagen accumulation in lung tissues, nearly devoid of tissue fibrosis. In contrast, the Bleomycin-treated rats displayed dense collagen deposition, extensive thickening of alveolar walls, and severe lung tissue fibrosis, particularly around blood vessels and in the peribronchiolar regions.

Conversely, Capsaicin treatment substantially reduced collagen deposition and accumulation when compared to the Bleomycin group. There was a notable reduction in observed fibrous tissue in the bronchiolar and alveolar regions as well as around blood vessels. This ameliorative effect was less pronounced in the Pirfenidone-treated group (Figure 2B). Staining with PAS to evaluate mucus secretion in the bronchiolar lumen revealed low mucus content in the control group. In contrast, the lung sections of Bleomycin-treated rats displayed a significant increase in mucus content. However, both Pirfenidone and Capsaicin administration reduced mucus content remarkably (Figure 2C).

### 3.4 The effects of Bleomycin, Pirfenidone, and Capsaicin on the pulmonary Myeloperoxidase (MPO) and hydroxyproline content of the treated rats

**FIGURE 3:**
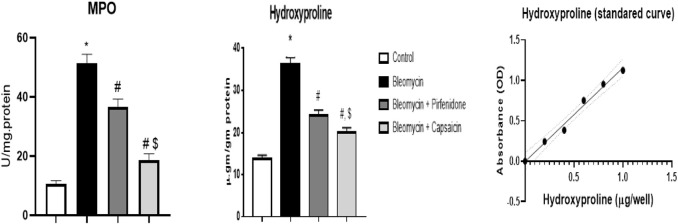
The effects of Bleomycin, Pirfenidone, and Capsaicin on the pulmonary hydroxyproline content. Bleomycin (5 mg/kg) was administered via intratracheal instillation on day zero. Capsaicin (3 mg/kg) and Pirfenidone (50 mg/kg) were orally administered daily for seven days. Results were analyzed by one-way ANOVA followed by Tukey’s as a post-hoc test (*n* = 8). *, #, and $ are considered statistically significant from the control, Bleomycin-only, and Bleomycin- plus Pirfenidone-treated group, respectively, at *p* < 0.05.

The data analysis revealed that Bleomycin administration resulted in a significant increase in the MPO and hydroxyproline content in the treated rats. Conversely, following the induction of lung fibrosis with Bleomycin, treatment with Pirfenidone and Capsaicin led to a significant reduction in both MPO and hydroxyproline contents compared to rats treated with Bleomycin alone. Capsaicin exhibited notably more promising results in this regard than Pirfenidone, as illustrated in Figure 3.

### 3.5 The effects of Bleomycin, Pirfenidone, and Capsaicin on the pulmonary oxidative stress biomarkers of the treated rats.

**FIGURE 4:**
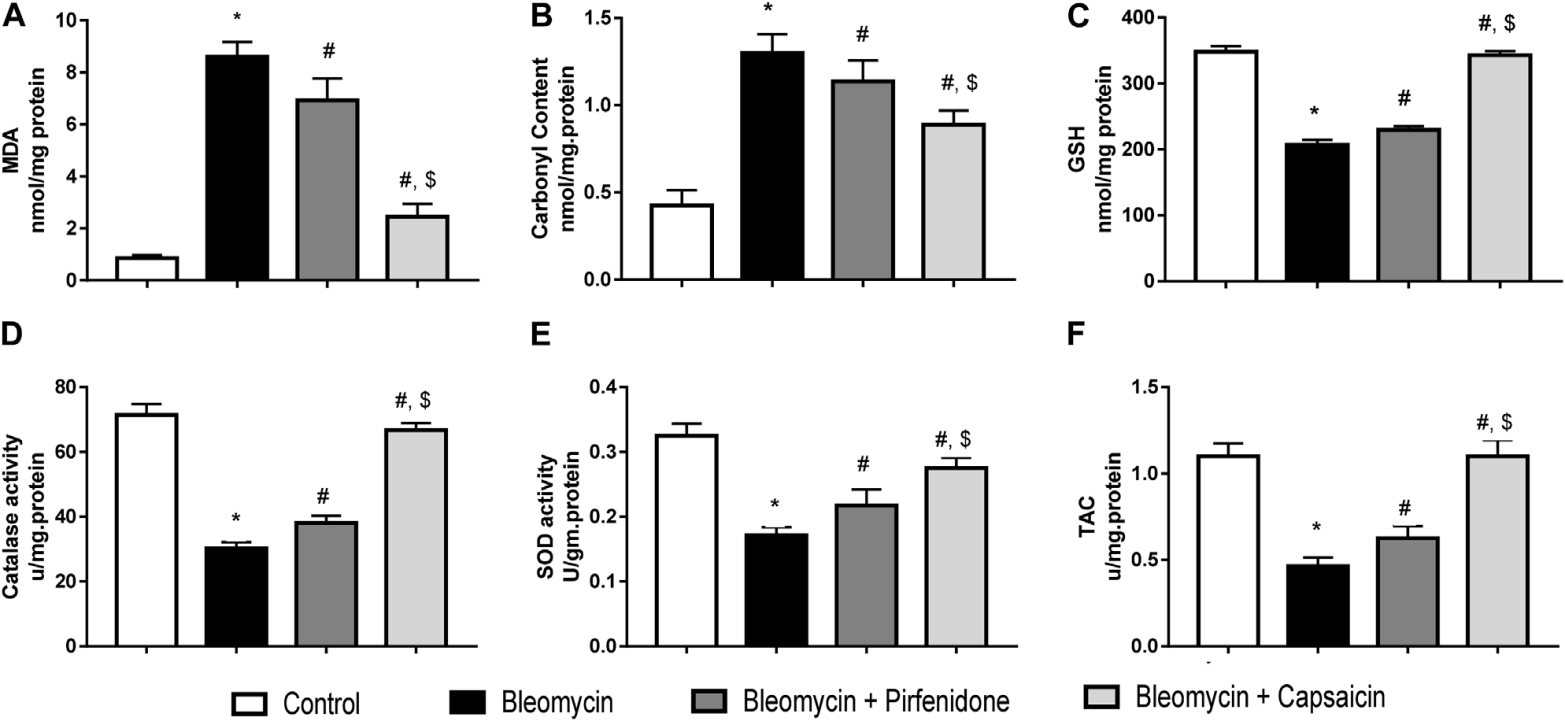
The effects of Bleomycin, Pirfenidone, and Capsaicin on the pulmonary malondialdehyde levels **(A)**, carbonyl content **(B)**, reduced glutathione levels **(C)**, catalase activity **(D)**, Superoxide dismutase activity **(E)**, and the total antioxidant capacity **(F)**. Bleomycin (5 mg/kg) was administered via intratracheal instillation on day zero. Capsaicin (3 mg/kg) and Pirfenidone (50 mg/kg) were orally administered daily for 7 days. Results were analyzed by one-way ANOVA followed by Tukey’s as a *post hoc* test (*n* = 8). *, #, and $ are considered statistically significant from the control, Bleomycin-only, and Bleomycin-plus Pirfenidone-treated group, respectively, at *p* < 0.05.

Data analyses revealed that a single intratracheal administration of Bleomycin induced oxidative stress, as evidenced by a significant increase in MDA levels and protein carbonyl content. Additionally, it led to a decrease in the levels of GSH, total antioxidant capacities, and the activities of both catalase and SOD when compared to the control group. However, when the Bleomycin-treated animals were challenged with either Pirfenidone or Capsaicin, these effects were significantly reversed. Notably, Capsaicin exhibited more potent antioxidant activity, with results comparable to those observed in the control animals, as depicted in Figure 4.

### 3.6 The effects of Bleomycin, Pirfenidone, and Capsaicin on the pulmonary NO levels

**FIGURE 5:**
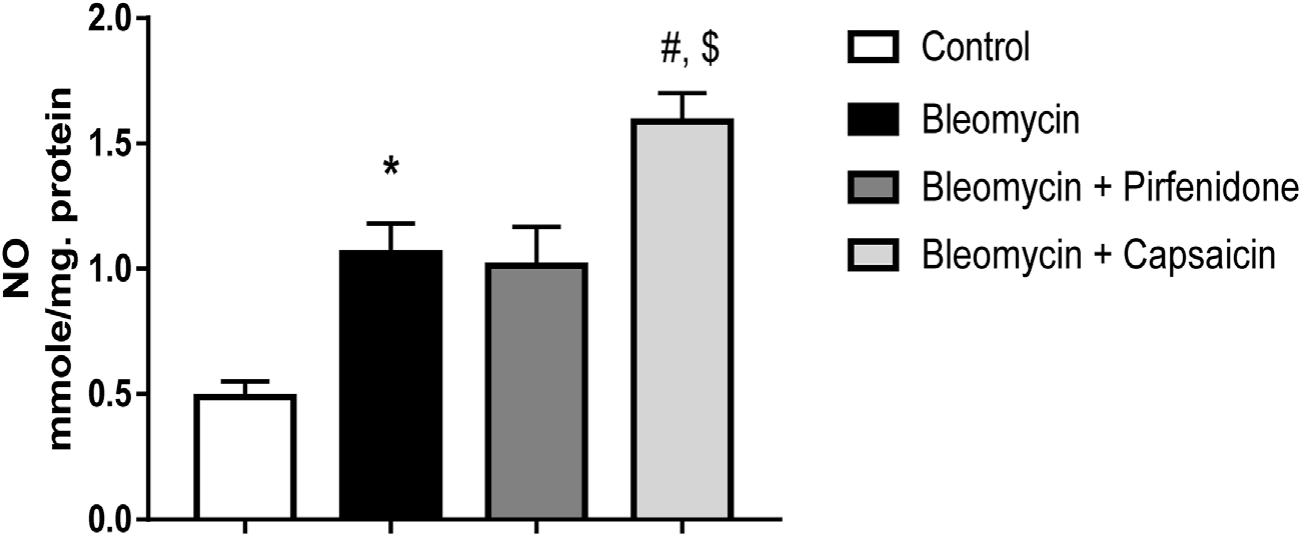
The effects of Bleomycin, Pirfenidone, and Capsaicin on the pulmonary nitric oxide (NO) levels. Bleomycin (5 mg/kg) was administered via intratracheal instillation on day zero. Capsaicin (3 mg/kg) and Pirfenidone (50 mg/kg) were orally administered daily for 7 days. Results were analyzed by one-way ANOVA followed by Tukey’s as a *post hoc* test (*n* = 8). *, #, and $ are considered statistically significant from the control, Bleomycin-only, and Bleomycin-plus Pirfenidone-treated group, respectively, at *p* < 0.05.

The administration of a single intratracheal dose of Bleomycin resulted in a notable increase in NO levels when compared to the intratracheal saline administration. Subsequent treatment of the Bleomycin-exposed animals with Pirfenidone did not lead to a significant decrease in NO levels. In contrast, the administration of Capsaicin significantly reduced the NO levels compared to the rats treated only with Bleomycin, as illustrated in Figure 5.

### 3.7 The effects of Bleomycin, Pirfenidone, and Capsaicin on the pulmonary TNF-α levels.

**FIGURE 6:**
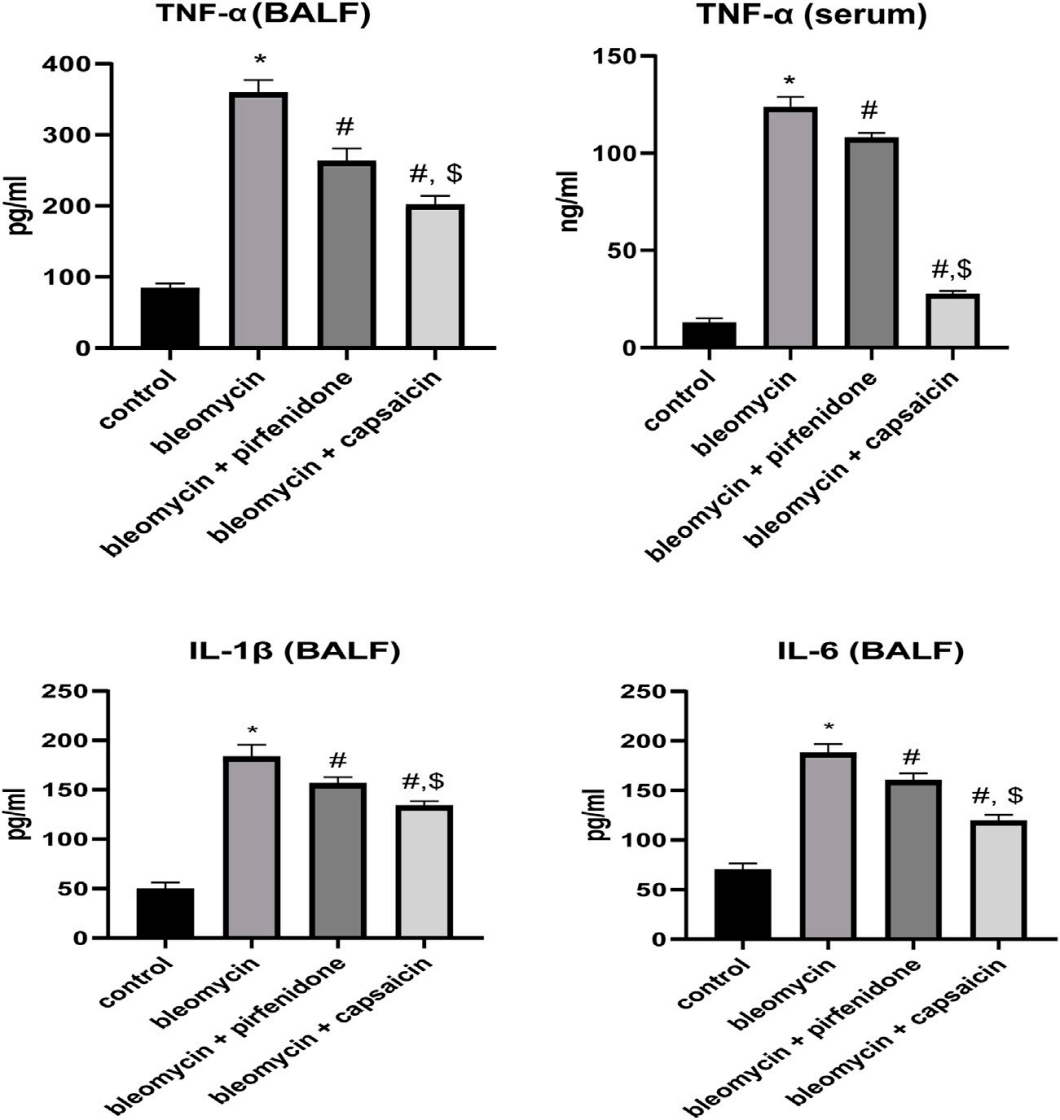
The effects of Bleomycin, Pirfenidone, and Capsaicin on the pulmonary tumor necrosis factor-alpha (TNF-α) levels in BALF and serum, IL-1β in BALF and IL-6 in BALF. Bleomycin (5 mg/kg) was administered via intratracheal instillation on day zero. Capsaicin (3 mg/kg) and Pirfenidone (50 mg/kg) were administered orally for 7 days. Results were analyzed by one-way ANOVA followed by Tukey’s as a *post hoc* test (*n* = 8). *, #, and $ are considered statistically significant from the control, Bleomycin-only, and Bleomycin-plus Pirfenidone-treated group, respectively, at *p* < 0.05.

The administration of Bleomycin resulted in a significant elevation in the protein levels of the inflammatory biomarker, TNF-α, compared to the levels in the saline-treated animals. However, treatment with Pirfenidone and Capsaicin significantly mitigated the increase in TNF-α levels induced by Bleomycin. Notably, Capsaicin’s effect in reducing the elevated TNF-α level was significantly more pronounced when compared to Pirfenidone, as depicted in Figure 6.

### 3.8 The effect of Bleomycin, Pirfenidone, and Capsaicin on the immunohistochemical reactivity of α-SMA, Nrf-2, NF-Kβ, COX 2, TGF-β1, and PPAR-γ in the rats’ lung tissue.

**FIGURE 7:**
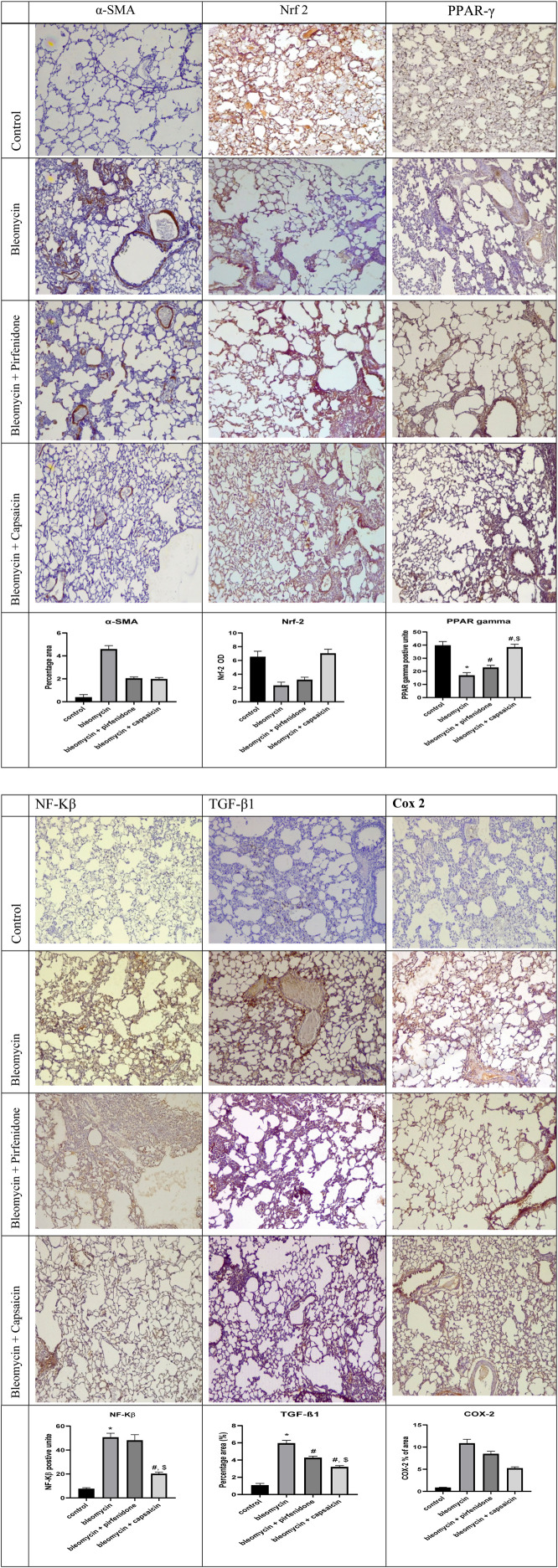
(Continued). The effects of Bleomycin, Pirfenidone, and Capsaicin on the pulmonary immunohistochemical reactivity of alpha-smooth muscle actin (α-SMA), nuclear factor erythroid 2–related factor 2 (Nrf-2), nuclear factor-kappa B (NF-Kβ), cyclooxygenase-2 (COX-2), transforming growth factor-beta 1 (TGF-β1), and Peroxisome proliferator-activated receptor gamma (PPAR-γ). Bleomycin (5 mg/kg) was administered via intratracheal instillation on day zero. Capsaicin (3 mg/kg) and Pirfenidone (50 mg/kg) were orally administered daily for 7 days. Results were analyzed by one-way ANOVA followed by Tukey’s as a *post hoc* test (*n* = 8). *, #, and $ are considered statistically significant from the control, Bleomycin-only, and Bleomycin-plus Pirfenidone-treated group, respectively, at *p* < 0.05.

Sections from rats treated solely with Bleomycin exhibited a significant increase in the immunoreactivity of α-SMA, NF-Kβ, COX 2, and TGF-β1. Furthermore, there was a significant decrease in the immunoreactivity of Nrf-2 and PPAR-γ compared to the control rats. Conversely, the administration of Pirfenidone significantly mitigated the impact of Bleomycin on α-SMA, Nrf-2, COX 2, TGF-β1, and PPAR-γ, although it did not significantly affect the immunoreactivity of NF-Kβ.Remarkably, Capsaicin administration substantially attenuated the effects of Bleomycin on the immunoreactivity of all the assessed parameters. Rats treated with Bleomycin and Capsaicin exhibited significantly reduced immunoreactivity of NF-Kβ and TGF-β1, along with increased immunoreactivity of PPAR-γ when compared to the rats treated with Bleomycin and Pirfenidone, as shown in Figure 7.

### 3.9 Western blot results

**FIGURE 8:**
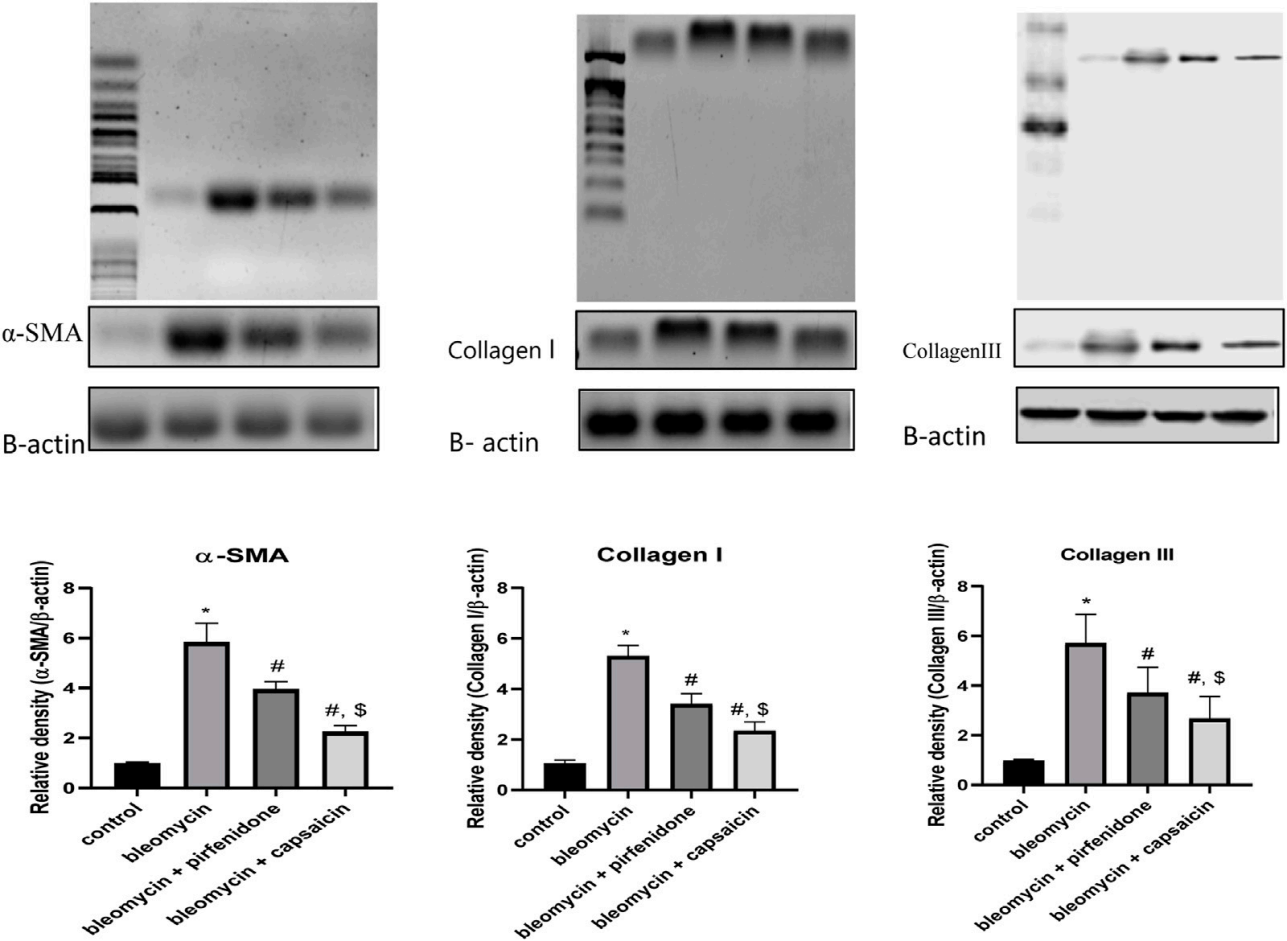
The effects of Bleomycin, Pirfenidone, and Capsaicin on the pulmonary protein expression of alpha-smooth muscle actin (α-SMA), CollagenI and CollagenIII. Results were analyzed by one-way ANOVA followed by Tukey’s as a *post hoc* test (*n* = 8). *, #, and $ are considered statistically significant from the control, Bleomycin-only, and Bleomycin-plus Pirfenidone-treated group, respectively, at *p* < 0.05.

In the present study, western blot technique was used to determine the levels of α-SMA, Collagen I and Collagen III in the pulmonary tissue, and it was found that both capsaicin and perfinidone administration could significantly ameliorate the increased levels of α-SMA, Collagen I and Collagen III proteins due to bleomycin intratracheal administration and the ameliorative effect of capsaicin was significantly higher than pirfenidone effect as shown in Figure 8.

## 4 Discussion

Bleomycin, a highly effective antibiotic with anticancer properties, but its therapeutic applications is limited since it has a tendency to induce lung fibrosis, which is depending on the dosage administered (Ezzie et al., 2011). Consequently, there has been a concerted effort to identify adjunct therapies that can mitigate this side effect. The present study represents the inaugural attempt to elucidate the therapeutic potential of Capsaicin in countering Bleomycin-induced pulmonary fibrosis.

The findings of this study underscore Bleomycin’s capacity to induce marked morphological and histopathological alterations in lung tissue, indicative of its potent fibrogenic effect. Macroscopic examination of the lungs revealed distinct manifestations of Bleomycin’s impact, including an irregular, coarse surface, interstitial hemorrhagic lesions, atrophic changes, tissue degeneration, significant tissue congestion, lung collapse, and tissue consolidation resulting in a loss of tissue elasticity. These observations align with prior research that has investigated the necroscopic effects of Bleomycin on lung tissues (Bahri et al., 2020). Such changes can be attributed to the inflammation, epithelial injury, and excessive extracellular deposition induced by Bleomycin (Birnhuber et al., 2020). At the microscopic level, Bleomycin led to tissue degeneration, alveolar collapse, thickening of inter-alveolar septa, extensive cellular infiltration, congested blood vessels, infiltration of lymphocytic cells, and shedding of bronchiolar epithelial cells. Additionally, there was an increase in collagen and mucin content. These features of bleomycin-induced pulmonary fibrosis were also evident in the previous studies (Demirkol et al., 2023; Gul et al., 2023).

Significantly, the outcomes of this study underscore the effectiveness of Capsaicin treatment in improving the macroscopic lung characteristics as well as the histopathological distortions induced by Bleomycin in a comparable and even more substantial restorative effect when compared to the commercially available antifibrotic drug, Pirfenidone. These effects can be attributed to the anti-inflammatory and antifibrotic properties of Capsaicin (Huang et al., 2022; Mansouri et al., 2023), which were further highlighted through the assessment of fibrotic and inflammatory markers.

Pulmonary fibrosis is a characteristic condition observed in patients undergoing Bleomycin treatment. The disruption of alveolar epithelial cells and subsequent alveolar collapse trigger the activation of fibroblasts, which engulf the collapsed alveoli and deposit collagen and other fibrotic proteins (Carneiro et al., 2017). The excessive deposition of collagen enhances the tensile strength of the pulmonary interstitium, contributing to the restrictive nature of fibrotic lung diseases (Danaei et al., 2023). Hydroxyproline is a major component of the fibrillar collagen, and it is increased in case of increased collagen production and decreased turnover after injury (Yang et al., 2023). Mucins, the most common glycoprotein components of mucus, are secreted into the extracellular space. Mucus can capture, retain, and release biologically active molecules such as cytokines and growth factors. At the same time, the secreted mucins can regulate inflammatory and immune responses (Schultz and Stick, 2015).

α-SMA serves as a crucial marker of lung fibrosis, where significant polymerization of α-SMA occurs in proliferating fibroblastic cells within the alveolar interstitium (Song et al., 2013; Wang et al., 2019). Another key mediator of fibrosis is TGF-β, a profibrotic cytokine that plays a central role in the induction of fibrogenesis. It is upregulated and activated during lung fibrosis and promotes myofibroblast activation, proliferation and trans-differentiation and matrix preservation (Liu et al., 2019). TGF-β1 binding to its receptor induces α-SMA expression and subsequent myofibroblast differentiation, resulting in increased collagen deposition, as explained by (Abbas et al., 2023).

Similarly, in the current study, intratracheal administration of Bleomycin elicited an increase in the fibrosis score according to the Ashcroft scoring system. Additionally, there was an evident rise in CollagenI and CollagenIII proteins expression as determined by western blot technique in addition to collagen and mucin pulmonary content, as demonstrated by MTC and PAS staining, respectively. These effects were associated with a significant increase in pulmonary fibrotic markers such as hydroxyproline, TGF-β1 and α-SMA.

Interestingly, Capsaicin administration demonstrated promising antifibrotic activity in lung tissue evidenced by the significant reduction in the fibrosis score, CollagenI and CollagenIII proteins expression, collagen deposition, mucin content, hydroxyproline content, and the immunohistochemical reactivity of α-SMA and TGF-β1, mirroring the effects of the approved antifibrotic Pirfenidone. Prior studies on Capsaicin have also substantiated its antifibrotic properties in various models, including its inhibition of TGF-β1 signaling to ameliorate kidney fibrosis (Liu et al., 2022).

To understand the mechanisms involved in the antifibrotic and ameliorative effects of Capsaicin in the Bleomycin-induced pulmonary fibrosis, we examined its impact on inflammatory cells besides some key mediators of inflammation and oxidative stress in the BAL and lung tissue.

Studies conducted on animal models involving intratracheal Bleomycin injection have revealed alterations in lavage fluid composition during fibrosis, characterized by a significant influx of inflammatory cells. Neutrophils are the first to appear, accompanied by increased percentages of lymphocytes and a transient influx of eosinophils, as documented by Fahey et al. (1982). Furthermore, Bleomycin-induced lung toxicity is marked by an augmentation in alveolar macrophages, as described by Kseibati et al. (2020). Protein molecules secreted by these inflammatory cells act as chemo-attractants and play a pivotal role in stimulating collagen synthesis by lung fibroblasts, contributing to lung fibrosis (Prasse et al., 2006).

The findings of the present study align with previous reports, wherein Bleomycin was found to increase both the total cell count and the percentages of macrophages, neutrophils, eosinophils, and lymphocytes. However, Capsaicin administration significantly mitigated the increase in the inflammatory cell count induced by Bleomycin. Capsaicin has previously demonstrated its ability to reduce inflammatory cell counts in BAL fluid in a model of lung ischemia-reperfusion injury in rabbits (Wang et al., 2019). These findings further underscore the anti-inflammatory effects elicited by Capsaicin.

The onset of pulmonary fibrosis is frequently preceded by acute inflammation of the lungs, triggered by viral and bacterial infections, radiation, chemotherapy, irritants, and pollutants. If left unresolved, this inflammation leads to the accumulation of fibrotic tissue in the lungs, causing respiratory dysfunction (Della Latta et al., 2015). Acute lung inflammation is characterized by the activation of inflammatory processes in response to lung injury, resulting in heightened permeability of lung capillary arteries and widespread destruction to the alveoli associated with fibrotic changes (Dorababu and Maraswami, 2023).

Upon lung injury, macrophages undergo a transformation into pro-inflammatory M1 phenotypes and start releasing pro-inflammatory cytokines (TNF-α, IL-6, IL-1β) and chemokines (IL-8, CCL7, CCL2). This results in an enhanced movement of monocytes and neutrophils towards the alveolar spaces, leading to their gradual accumulation (Brill et al., 2015; Frey et al., 2021). Neutrophils, in response, secrete a multitude of inflammatory mediators, reactive oxygen species, and proteinases, which cause damage to surfactant, basal membranes, and the epithelia-endothelial barrier, eventually leading to pulmonary fibrosis. Surfactant is a lipid-protein complex produced by alveolar epithelial type II cells (Dhooghe et al., 2014). It acts to reduce surface tension in the alveoli, preventing their collapse. During the development of acute lung injury, the loss of type II alveolar epithelial cells (AEC II) causes a notable reduction in surfactant production. Consequently, the collapse of alveoli occurs, allowing lung proteins to permeate into the alveolar space (Carignon et al., 2023; Ye et al., 2023).

The possible mechanisms of pulmonary tissue damage, fibrosis and cell death is disruption of tight-junction mediated cell-to-cell contacts, changes in extracellular matrix components and their interaction with epithelial cells, and disturbances in communication between epithelial and immune cells (Della Latta et al., 2015; Evangelista-Leite et al., 2023).

The current study revealed that Bleomycin increased the expression of inflammatory mediators such as TNF-α (BALF and lung tissue), NF-κB, TGF-β, IL-6 and Il-β along with elevated expression of the inducible COX-2 and increased NO production. Whereas, prostaglandins produced due to increased COX-2 expression have also been associated with the acceleration of pulmonary fibrosis (Oga et al., 2009).

Capsaicin administration effectively counteracted the inflammatory effects induced by Bleomycin by reducing the expression of TNF-α, NF-κB, TGF-β1 and COX-2, as well as decreasing NO production. These results align with previous studies demonstrating Capsaicin’s anti-inflammatory effects against cyclophosphamide and acrylamide toxicities, as elucidated by Melekoglu et al. (2018); Abd Al Haleem et al. (2022).

Bleomycin was observed to decrease the immunoreactivity of PPAR-γ, a member of the nuclear hormone receptor family known for its role in modulating immune responses. PPAR-γ inhibits the expression of inflammatory mediators and influences immune cell differentiation toward anti-inflammatory phenotypes (Pascual et al., 2005). Additionally, PPAR-γ has been found to inhibit fibroblast proliferation, induce cell cycle arrest, and impede TGF-β1-induced myofibroblast differentiation and collagen secretion, hence effectively inhibit fibrosis (Milam et al., 2008). These findings suggest the potential of PPAR-γ agonists as therapeutic agents against pulmonary fibrosis. Interestingly, the present study demonstrated that capsaicin increased the expression of PPAR-γ in lung tissue, thereby we may postulate that the antifibrotic and anti-inflammatory properties of Capsaicin may be mediated in part through PPAR-γ activation.

The initial cellular injury accompanying lung fibrosis is mediated by reactive oxygen species (ROS) produced by infiltrating inflammatory cells. Activated macrophages, for instance, produce NO and peroxynitrite (Barnes and Belvisi, 1993). Moreover, inflammation and oxidative stress are interrelated, as NF-kB is activated in response to ROS production (Sen and Packer, 1996). Additionally, activated inflammatory cells produce ROS, which, in turn, recruit other inflammatory cells and amplifying the damage (Nathan, 2003). Nuclear factor erythroid 2–related factor 2 (Nrf-2) is a master regulator of cellular resistance to oxidative stress via modulation of the expression of genes encoding antioxidant enzymes and genes controlling immune and inflammatory responses, tissue remodeling and fibrosis (Hao et al., 2022). Alterations in the redox state activate Nrf2 which upregulates the production of antioxidant, xenobiotic-metabolizing, and cytoprotective enzymes, safeguarding cells against ROS (Chan and Kan, 1999). In the present study, Bleomycin significantly induced oxidative stress with increased ROS production, upregulated MPO pulmonary tissue concentration, elevated NO levels, elevated lipid peroxidation and protein oxidation products (MDA and protein carbonyls respectively), decreased GSH levels, and total antioxidant capacity and reduced activity of antioxidant enzymes (catalase and SOD). This disturbance in the oxidative balance was associated with downregulation Nrf-2. Similar findings were reported by previous studies (Han et al., 2019), Capsaicin administration markedly alleviated oxidative stress by upregulating Nrf-2 expression, increasing antioxidant enzyme activities, GSH levels, and total antioxidant capacity, and reducing the products of oxidative stress, including MDA and protein carbonyls. Our results were in accordance with several studies highlighting the antioxidant effects of Capsaicin (Beltran et al., 2007; Nascimento et al., 2014; Thongin et al., 2022).

Collectively, Capsaicin exhibited promising antifibrotic activity against Bleomycin-induced pulmonary fibrosis and demonstrating efficacy comparable to the commercially available and approved antifibrotic drug, Pirfenidone. These antifibrotic effects may be attributed, at least in part, to the antioxidant and anti-inflammatory activities of Capsaicin mediated by upregulation of PPAR-γ and Nrf-2 expression.

## 5 Study limitations

Moving forward, there are several promising avenues for further research and clinical applications regarding Capsaicin’s potential in treating pulmonary fibrosis. Firstly, deeper investigations into the precise molecular mechanisms underlying Capsaicin’s antifibrotic and anti-inflammatory effects are warranted. Understanding how Capsaicin interacts with specific signaling pathways involved in fibrosis, such as TGF-β1, PPAR-γ, and NF-κB, can provide valuable insights and potential targets for drug development. Additionally, optimizing the dosage and administration regimen of Capsaicin is crucial. Future studies should explore various dosages and treatment durations to determine the most effective and safe therapeutic regimen. This will help establish standardized protocols for clinical applications. Combination therapies could hold great promise in enhancing treatment outcomes. Exploring the potential synergies between Capsaicin and existing medications like Pirfenidone or Nintedanib may lead to more potent treatment strategies for pulmonary fibrosis. Transitioning from preclinical studies to clinical trials is a critical step. Well-designed clinical trials are needed to evaluate the safety and efficacy of Capsaicin in patients with pulmonary fibrosis. These trials should assess parameters such as lung function, quality of life, and fibrosis progression. Identifying specific biomarkers associated with Capsaicin’s therapeutic effects can aid in patient selection and monitoring of treatment responses, allowing for more personalized treatment approaches.

## 6 Conclusion

This study sheds light on the potential therapeutic efficacy of Capsaicin in mitigating the detrimental effects of Bleomycin which is an effective anticancer agent with high potential to cause dose-dependent pulmonary fibrosis. Our findings reveal that Capsaicin treatment exerts a considerable positive impact on both the macroscopic and microscopic aspects of lung tissue affected by Bleomycin. Intratracheal administration of bleomycin produced visible signs of fibrosis, histopathological alterations, increased collagen deposition, elevated mucin content, inflammatory cell infiltration and elevated fibrosis markers such as hydroxyproline, α-SMA and TGF-β1. Inflammatory markers such a TNF-α, IL-1β, IL-6, NF-κB and COX-2 as well as oxidative stress markers such as NO, MDA, and protein carbonyl were induced by bleomycin. Moreover, anti-inflammatory and antoioxidant mechanisms were compromised by bleomycin as evident by decreased the expression of PPAR-γ and Nrf-2, reduced GSH, total antioxidant capacity, and the activities of catalase and SOD.

On the other hand, treatment with Capsaicin following Bleomycin exposure improved lung macroscopic and microscopic characteristics and reversed the histopathological distortions induced by Bleomycin compared to the approved antifibrotic drug Pirfenidone. These effects were evidenced in the form of reduced collagen deposition, fibrosis score and mucin content, decreased inflammatory cell infiltration, lowered levels of fibrosis markers (hydroxyproline, α-SMA, and TGF-β1), downregulated inflammatory markers (TNF-α, IL-1β, IL-6, NF-κB, and COX-2) and oxidative stress markers (NO, MDA, and protein carbonyl). Parallel to these effects, Capsaicine enhanced the anti-inflammatory and antioxidant pathways (PPAR-γ, Nrf-2, GSH, total antioxidant capacity, and the activities of catalase and SOD.

**In summary**, Bleomycin-induced pulmonary fibrosis is closely associated with inflammatory responses and oxidative stress. Capsaicin emerges as a promising candidate for the treatment of Bleomycin-induced pulmonary fibrosis, with its multifaceted benefits encompassing anti-inflammatory, antifibrotic, and antioxidant activities. These findings pave the way for further research and clinical investigations and potentially offer new therapeutic avenues for managing pulmonary fibrosis and enhancing the quality of life for affected individuals.

## Data Availability

The original contributions presented in the study are included in the article/Supplementary Material, further inquiries can be directed to the corresponding author.

## References

[B1] Abbas N. A. T. Nafea O. E. Mohammed H. O. Samy W. Abdelmageed A. F. Afifi R. (2023). Repurposing of carvedilol to alleviate bleomycin-induced lung fibrosis in rats: repressing of TGF-β1/α-SMA/Smad2/3 and STAT3 gene expressions. Life Sci. 324, 121692. 10.1016/j.lfs.2023.121692 37061127

[B2] Abd Al Haleem E. N. Hasan W. Y. S. Arafa H. M. M. (2022). Therapeutic effects of thymoquinone or capsaicin on acrylamide-induced reproductive toxicity in rats mediated by their effect on oxidative stress, inflammation, and tight junction integrity. Drug Chem. Toxicol. 45 (5), 2328–2340. 10.1080/01480545.2021.1942485 34233550

[B3] Altintas N. Erboga M. Aktas C. Bilir B. Aydin M. Sengul A. (2016). Protective effect of infliximab, a tumor necrosis factor-alfa inhibitor, on bleomycin-induced lung fibrosis in rats. Inflammation 39 (1), 65–78. 10.1007/s10753-015-0224-z 26253295

[B4] Archer S. (1993). Measurement of nitric oxide in biological models. Faseb J. 7 (2), 349–360. 10.1096/fasebj.7.2.8440411 8440411

[B5] Ayilya B. L. Balde A. Ramya M. Benjakul S. Kim S. K. Nazeer R. A. (2023). Insights on the mechanism of bleomycin to induce lung injury and associated *in vivo* models: a review. Int. Immunopharmacol. 121, 110493. 10.1016/j.intimp.2023.110493 37331299

[B6] Bahri S. Ben Ali R. Nahdi A. Mlika M. Abdennabi R. Jameleddine S. (2020). Salvia officinalis attenuates bleomycin-induced oxidative stress and lung fibrosis in rats. Nutr. Cancer 72 (7), 1135–1145. 10.1080/01635581.2019.1675724 31608667

[B7] Barnes P. J. Belvisi M. J. T. (1993). Nitric oxide and lung disease. Thorax 48 (10), 1034–1043. 10.1136/thx.48.10.1034 7903007 PMC464825

[B8] Beltran J. Ghosh A. K. Basu S. (2007). Immunotherapy of tumors with neuroimmune ligand capsaicin. J. Immunol. 178 (5), 3260–3264. 10.4049/jimmunol.178.5.3260 17312175

[B9] Birnhuber A. Egemnazarov B. Biasin V. Bonyadi Rad E. Wygrecka M. Olschewski H. (2020). CDK4/6 inhibition enhances pulmonary inflammatory infiltration in bleomycin-induced lung fibrosis. Respir. Res. 21 (1), 167. 10.1186/s12931-020-01433-w 32616042 PMC7331186

[B10] Brill S. E. Patel A. R. C. Singh R. Mackay A. J. Brown J. S. Hurst J. R. (2015). Lung function, symptoms and inflammation during exacerbations of non-cystic fibrosis bronchiectasis: a prospective observational cohort study. Respir. Res. 16 (1), 16. 10.1186/s12931-015-0167-9 25849856 PMC4324878

[B11] Carignon S. De Moura Rodrigues D. Gosset D. Culerier E. Huot-Marchand S. Savigny F. (2023). Lung inflammation and interstitial fibrosis by targeted alveolar epithelial type I cell death. Front. Immunol. 14, 1261483. 10.3389/fimmu.2023.1261483 37841243 PMC10568624

[B12] Carneiro P. J. Clevelario A. L. Padilha G. A. Silva J. D. Kitoko J. Z. Olsen P. C. (2017). Bosutinib therapy ameliorates lung inflammation and fibrosis in experimental silicosis. Front. Physiol. 8, 159. 10.3389/fphys.2017.00159 28360865 PMC5350127

[B13] Chan K. Kan Y. W. (1999). Nrf2 is essential for protection against acute pulmonary injury in mice. Proc. Natl. Acad. Sci. U. S. A. 96 (22), 12731–12736. 10.1073/pnas.96.22.12731 10535991 PMC23072

[B14] Chen W. Pilling D. Gomer R. H. (2023). The mRNA-binding protein DDX3 mediates TGF-β1 upregulation of translation and promotes pulmonary fibrosis. JCI Insight 8 (7), e167566. 10.1172/jci.insight.167566 36821384 PMC10132153

[B15] Cissell D. D. Link J. M. Hu J. C. Athanasiou K. A. (2017). A modified hydroxyproline assay based on hydrochloric acid in Ehrlich's solution accurately measures tissue collagen content. Tissue Eng. Part C Methods 23 (4), 243–250. 10.1089/ten.tec.2017.0018 28406755 PMC5397204

[B16] Danaei N. Sadeghi H. Asfarm A. Rostamzadeh D. Panahi Kokhdan E. Sadeghi H. (2023). Betulin-rich hydroalcoholic extract of Daphne oleoides attenuates bleomycin-induced pulmonary fibrosis in rat. Heliyon 9 (8), e19236. 10.1016/j.heliyon.2023.e19236 37664747 PMC10469556

[B17] Della Latta V. Cecchettini A. Del Ry S. Morales M. A. (2015). Bleomycin in the setting of lung fibrosis induction: from biological mechanisms to counteractions. Pharmacol. Res. 97, 122–130. 10.1016/j.phrs.2015.04.012 25959210

[B18] Demirkol B. Gul S. Cörtük M. Akanıl Fener N. Yavuzsan E. Eren R. (2023). Protective efficacy of pirfenidone in rats with pulmonary fibrosis induced by bleomycin. Sarcoidosis Vasc. Diffuse Lung Dis. 40 (3), e2023036. 10.36141/svdld.v40i3.13847 37712376 PMC10540724

[B19] Dhooghe B. Noël S. Huaux F. Leal T. (2014). Lung inflammation in cystic fibrosis: pathogenesis and novel therapies. Clin. Biochem. 47 (7-8), 539–546. 10.1016/j.clinbiochem.2013.12.020 24380764

[B20] Dorababu A. Maraswami M. (2023). Recent advances (2015-2020) in drug discovery for attenuation of pulmonary fibrosis and COPD. Molecules 28 (9), 3674. 10.3390/molecules28093674 37175084 PMC10179756

[B21] Evangelista-Leite D. Carreira A. C. O. Nishiyama M. Y. Gilpin S. E. Miglino M. A. (2023). The molecular mechanisms of extracellular matrix-derived hydrogel therapy in idiopathic pulmonary fibrosis models. Biomaterials 302, 122338. 10.1016/j.biomaterials.2023.122338 37820517

[B22] Ezzie M. E. Piper M. G. Montague C. Newland C. A. Opalek J. M. Baran C. (2011). Thrombospondin-1-deficient mice are not protected from bleomycin-induced pulmonary fibrosis. Am. J. Respir. Cell Mol. Biol. 44 (4), 556–561. 10.1165/rcmb.2009-0019OC 20581099 PMC3095927

[B60] Fahey P. J. (1982). Early diagnosis of bleomycin pulmonary toxicity using bronchoalveolar lavage in dogs. Am. Rev. Respir. Dis. 126 (1), 126–30. 6178329 10.1164/arrd.1982.126.1.126

[B23] Frey D. L. Boutin S. Dittrich S. A. Graeber S. Y. Stahl M. Wege S. (2021). Relationship between airway dysbiosis, inflammation and lung function in adults with cystic fibrosis. J. Cyst. Fibros. 20 (5), 754–760. 10.1016/j.jcf.2020.12.022 33431308

[B24] Gul A. Yang F. Xie C. Du W. Mohammadtursun N. Wang B. (2023). Pulmonary fibrosis model of mice induced by different administration methods of bleomycin. BMC Pulm. Med. 23 (1), 91. 10.1186/s12890-023-02349-z 36944966 PMC10029181

[B25] Guo J. Yang Z. Jia Q. Bo C. Shao H. Zhang Z. (2019). Pirfenidone inhibits epithelial-mesenchymal transition and pulmonary fibrosis in the rat silicosis model. Toxicol. Lett. 300, 59–66. 10.1016/j.toxlet.2018.10.019 30394303

[B26] Han B. Li S. Lv Y. Yang D. Li J. Yang Q. (2019). Dietary melatonin attenuates chromium-induced lung injury via activating the Sirt1/Pgc-1α/Nrf2 pathway. Food Funct. 10 (9), 5555–5565. 10.1039/c9fo01152h 31429458

[B27] Hao W. Li M. Cai Q. Wu S. Li X. He Q. (2022). Roles of NRF2 in fibrotic diseases: from mechanisms to therapeutic approaches. Front. Physiol. 13, 889792. 10.3389/fphys.2022.889792 35721561 PMC9203969

[B28] Henderson R. F. J. E. Pathology T. (2005). Use of bronchoalveolar lavage to detect respiratory tract toxicity of inhaled material. Exp. Toxicol. Pathol. 57, 155–159. 10.1016/j.etp.2005.05.004 16092723

[B29] Huang Z. Sharma M. Dave A. Yang Y. Chen Z. S. Radhakrishnan R. (2022). The antifibrotic and the anticarcinogenic activity of capsaicin in hot chili pepper in relation to oral submucous fibrosis. Front. Pharmacol. 13, 888280. 10.3389/fphar.2022.888280 35600864 PMC9114457

[B30] Ilie M. A. Caruntu C. Tampa M. Georgescu S. R. Matei C. Negrei C. (2019). Capsaicin: physicochemical properties, cutaneous reactions and potential applications in painful and inflammatory conditions. Exp. Ther. Med. 18 (2), 916–925. 10.3892/etm.2019.7513 31384324 PMC6639979

[B31] Ishida Y. Mabuchi Y. Naraoka Y. Hisamatsu D. Akazawa C. (2023). Conservation of markers and stemness in adipose stem and progenitor cells between cattle and other species. Int. J. Mol. Sci. 24 (4), 11908. 10.3390/ijms241511908 37569284 PMC10418360

[B32] Kabel A. M. Omar M. S. Elmaaboud M. A. A. (2016). Amelioration of bleomycin-induced lung fibrosis in rats by valproic acid and butyrate: role of nuclear factor kappa-B, proinflammatory cytokines and oxidative stress. Int. Immunopharmacol. 39, 335–342. 10.1016/j.intimp.2016.08.008 27526269

[B33] Kadam A. H. Schnitzer J. E. (2023). Characterization of acute lung injury in the bleomycin rat model. Physiol. Rep. 11 (5), e15618. 10.14814/phy2.15618 36898724 PMC10005890

[B34] Kseibati M. O. Shehatou G. S. G. Sharawy M. H. Eladl A. E. Salem H. A. (2020). Nicorandil ameliorates bleomycin-induced pulmonary fibrosis in rats through modulating eNOS, iNOS, TXNIP and HIF-1α levels. Life Sci. 246, 117423. 10.1016/j.lfs.2020.117423 32057902

[B35] Lee C. Y. Kim M. Yoon S. W. (2003). Short-term control of capsaicin on blood and oxidative stress of rats *in vivo* . Phytother. Res. 17 (5), 454–458. 10.1002/ptr.1172 12748978

[B36] Lei L. Zhao C. Qin F. He Z. Y. Wang X. Zhong X. N. (2016). Th17 cells and IL-17 promote the skin and lung inflammation and fibrosis process in a bleomycin-induced murine model of systemic sclerosis. Clin. Exp. Rheumatol. 34 (5), 14–22. 26750756

[B37] Liu B. Bing Q. Li S. Han B. Lu J. Baiyun R. (2019). Role of A(2B) adenosine receptor-dependent adenosine signaling in multi-walled carbon nanotube-triggered lung fibrosis in mice. J. Nanobiotechnology 17 (1), 45. 10.1186/s12951-019-0478-y 30922349 PMC6440149

[B38] Liu Z. Wang W. Li X. Tang S. Meng D. Xia W. (2022). Capsaicin ameliorates renal fibrosis by inhibiting TGF-β1–Smad2/3 signaling. Phytomedicine 100, 154067. 10.1016/j.phymed.2022.154067 35349832

[B39] Mansouri R. A. Ahmed A. M. Alshaibi H. F. Aboubakr E. M. (2023). Capsaicin ameliorates myocardial injury in diabetic rats via upregulating Nrf-2, HO-1 and iNOS tissue concentrations besides normalizing the distribution of structural proteins desmin and α-SMA. Food Biosci. 56, 103130. 10.1016/j.fbio.2023.103130

[B40] Melekoglu R. Ciftci O. Eraslan S. Cetin A. Basak N. (2018). Beneficial effects of curcumin and capsaicin on cyclophosphamide-induced premature ovarian failure in a rat model. J. Ovarian Res. 11 (1), 33–38. 10.1186/s13048-018-0409-9 29699594 PMC5918567

[B41] Milam J. E. Keshamouni V. G. Phan S. H. Hu B. Gangireddy S. R. Hogaboam C. M. (2008). PPAR-γ agonists inhibit profibrotic phenotypes in human lung fibroblasts and bleomycin-induced pulmonary fibrosis. Am. J. Physiol. Lung Cell. Mol. Physiol. 294 (5), L891–L901. 10.1152/ajplung.00333.2007 18162602 PMC5926773

[B42] Nascimento P. L. Nascimento T. C. E. S. Ramos N. S. M. Silva G. R. Gomes J. E. G. Falcão R. E. A. (2014). Quantification, antioxidant and antimicrobial activity of phenolics isolated from different extracts of Capsicum frutescens (Pimenta Malagueta). Molecules 19 (4), 5434–5447. 10.3390/molecules19045434 24879587 PMC6271728

[B43] Nathan C. (2003). Specificity of a third kind: reactive oxygen and nitrogen intermediates in cell signaling. J. Clin. Invest. 111 (6), 769–778. 10.1172/JCI18174 12639979 PMC153776

[B44] Oga T. Matsuoka T. Yao C. Nonomura K. Kitaoka S. Sakata D. (2009). Prostaglandin F(2alpha) receptor signaling facilitates bleomycin-induced pulmonary fibrosis independently of transforming growth factor-beta. Nat. Med. 15 (12), 1426–1430. 10.1038/nm.2066 19966781

[B45] Pascual G. Fong A. L. Ogawa S. Gamliel A. Li A. C. Perissi V. (2005). A SUMOylation-dependent pathway mediates transrepression of inflammatory response genes by PPAR-gamma. Nature 437 (7059), 759–763. 10.1038/nature03988 16127449 PMC1464798

[B46] Prasse A. Pechkovsky D. V. Toews G. B. Jungraithmayr W. Kollert F. Goldmann T. (2006). A vicious circle of alveolar macrophages and fibroblasts perpetuates pulmonary fibrosis via CCL18. Am. J. Respir. Crit. Care Med. 173 (7), 781–792. 10.1164/rccm.200509-1518OC 16415274

[B47] Razzaque M. S. Hossain M. A. Kohno S. Taguchi T. (1998). Bleomycin-induced pulmonary fibrosis in rat is associated with increased expression of collagen-binding heat shock protein (HSP) 47. Virchows Arch. 432 (5), 455–460. 10.1007/s004280050191 9645446

[B48] Schultz A. Stick S. (2015). Early pulmonary inflammation and lung damage in children with cystic fibrosis. Respirology 20 (4), 569–578. 10.1111/resp.12521 25823858

[B49] Sen C. K. Packer L. J. (1996). Antioxidant and redox regulation of gene transcription. FASEB J. 10 (7), 709–720. 10.1096/fasebj.10.7.8635688 8635688

[B50] Song X. Liu W. Xie S. Wang M. Cao G. Mao C. (2013). All-transretinoic acid ameliorates bleomycin-induced lung fibrosis by downregulating the TGF-β1/Smad3 signaling pathway in rats. Lab. Invest. 93 (11), 1219–1231. 10.1038/labinvest.2013.108 24042439

[B51] Thongin S. Den-Udom T. Uppakara K. Sriwantana T. Sibmooh N. Laolob T. (2022). Beneficial effects of capsaicin and dihydrocapsaicin on endothelial inflammation, nitric oxide production and antioxidant activity. Biomed. Pharmacother. 154, 113521. 10.1016/j.biopha.2022.113521 36007275

[B52] Usman M. Faruqui Z. S. ud Din N. Zahid K. F. (2010). Bleomycin induced pulmonary toxicity in patients with germ cell tumours. J. Ayub Med. Coll. Abbottabad 22 (3), 35–37. 22338413

[B53] Venkatesan N. Ouzzine M. Kolb M. Netter P. Ludwig M. S. (2011). Increased deposition of chondroitin/dermatan sulfate glycosaminoglycan and upregulation of β1,3-glucuronosyltransferase I in pulmonary fibrosis. Am. J. Physiol. Lung Cell Mol. Physiol. 300 (2), L191–L203. 10.1152/ajplung.00214.2010 21056957

[B54] Wang H. H. Meng Y. L. Yang Z. M. Wang X. X. Xu H. X. Wang W. M. (2019). Effect of Dilong on expression of fibrogenic factors TGF-β1 and α-SMA in lung tissue of mice with pulmonary fibrosis. Zhongguo Zhong Yao Za Zhi 44 (24), 5473–5478. 10.19540/j.cnki.cjcmm.20190716.402 32237397

[B55] Wang Q. Sundar I. K. Lucas J. H. Park J. G. Nogales A. Martinez-Sobrido L. (2023). Circadian clock molecule REV-ERBα regulates lung fibrotic progression through collagen stabilization. Nat. Commun. 14 (1), 1295. 10.1038/s41467-023-36896-0 36894533 PMC9996598

[B56] Yang X. Huang X. J. Chen Z. Xu A. L. Zhou H. Bi X. L. (2023). A novel quantification method of lung fibrosis based on Micro-CT images developed with the optimized pulmonary fibrosis mice model induced by bleomycin. Heliyon 9 (3), e13598. 10.1016/j.heliyon.2023.e13598 36895392 PMC9988492

[B57] Ye X. Zhang M. Gu H. Liu M. Zhao Y. Shi Y. (2023). Animal models of acute exacerbation of pulmonary fibrosis. Respir. Res. 24 (1), 296. 10.1186/s12931-023-02595-z 38007420 PMC10675932

[B58] Yoshizaki A. Yanaba K. Iwata Y. Komura K. Ogawa A. Akiyama Y. (2010). Cell adhesion molecules regulate fibrotic process via Th1/Th2/Th17 cell balance in a bleomycin-induced scleroderma model. J. Immunol. 185 (4), 2502–2515. 10.4049/jimmunol.0901778 20624949 PMC3733122

[B59] Zimmer A. R. Leonardi B. Miron D. Schapoval E. Oliveira J. R. D. Gosmann G. (2012). Antioxidant and anti-inflammatory properties of Capsicum baccatum: from traditional use to scientific approach. J. Ethnopharmacol. 139 (1), 228–233. 10.1016/j.jep.2011.11.005 22100562

