# Supplementary material for: Capsaicin ameliorate pulmonary fibrosis via antioxidant Nrf-2/ PPAR- γ pathway activation and inflammatory TGF-β1/ NF-κB/COX II pathway inhibition
Source: Front Pharmacol. 2024 Feb 21;15:1333715. doi: 10.3389/fphar.2024.1333715 (PMC10915016; doi:10.3389/fphar.2024.1333715)
Supplement: Supplementary file 1 [file Table1.DOCX]

**Alpha SMA**


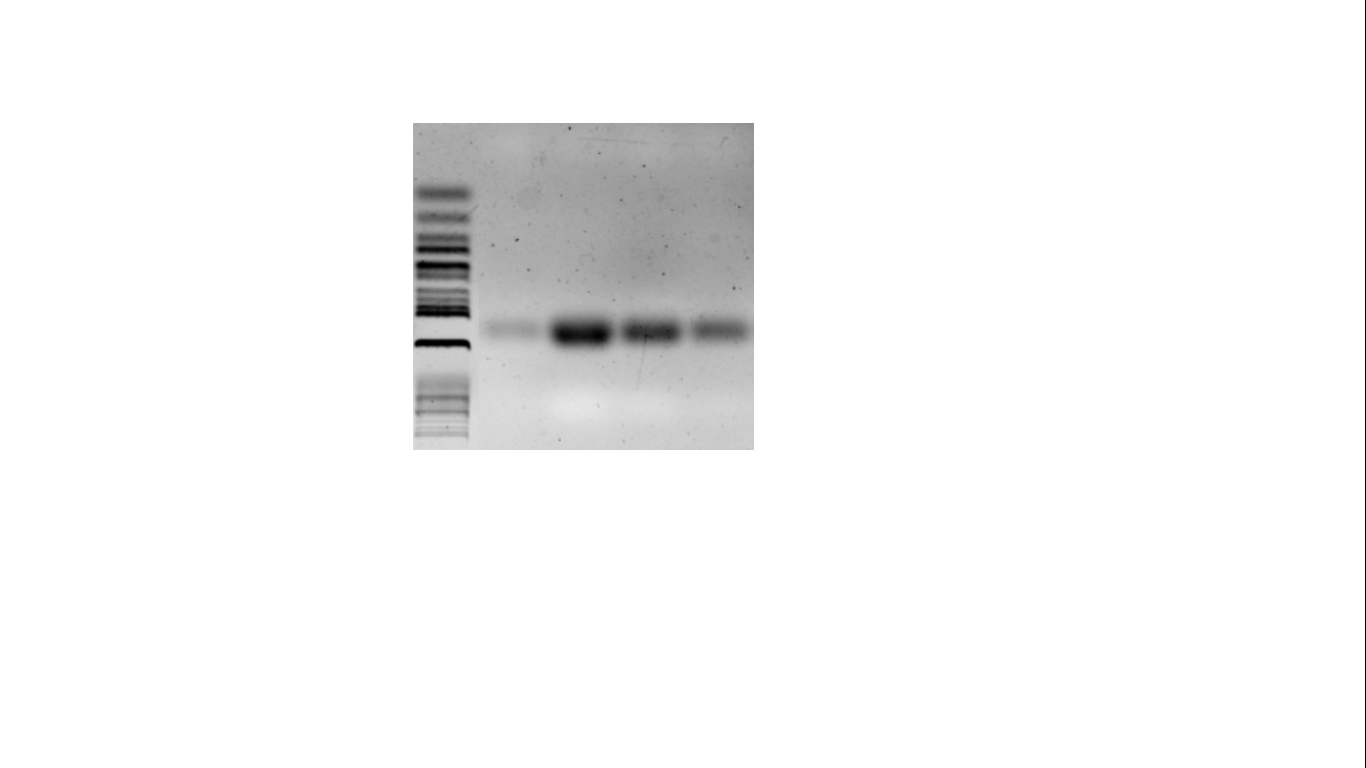
**Repeat 1**

**
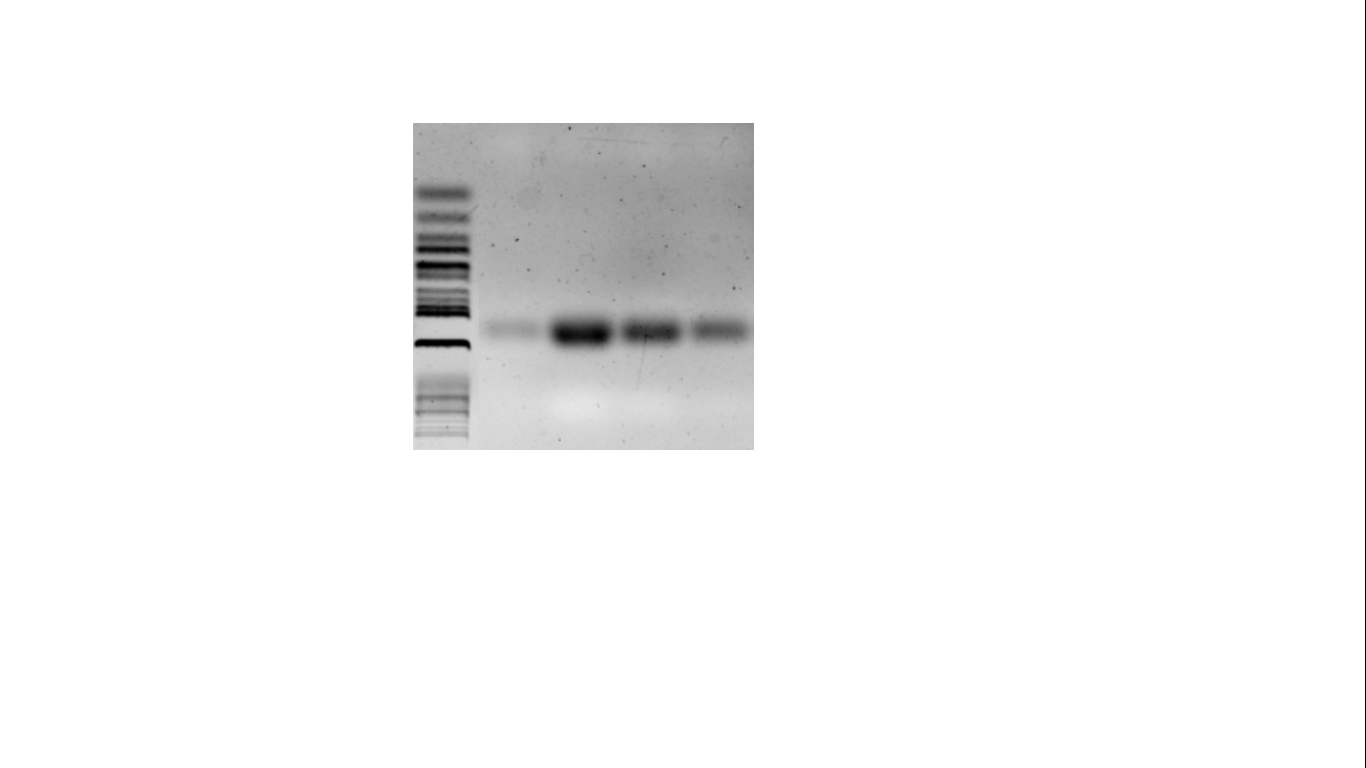
**

ᵅ-SMA 42 KDa


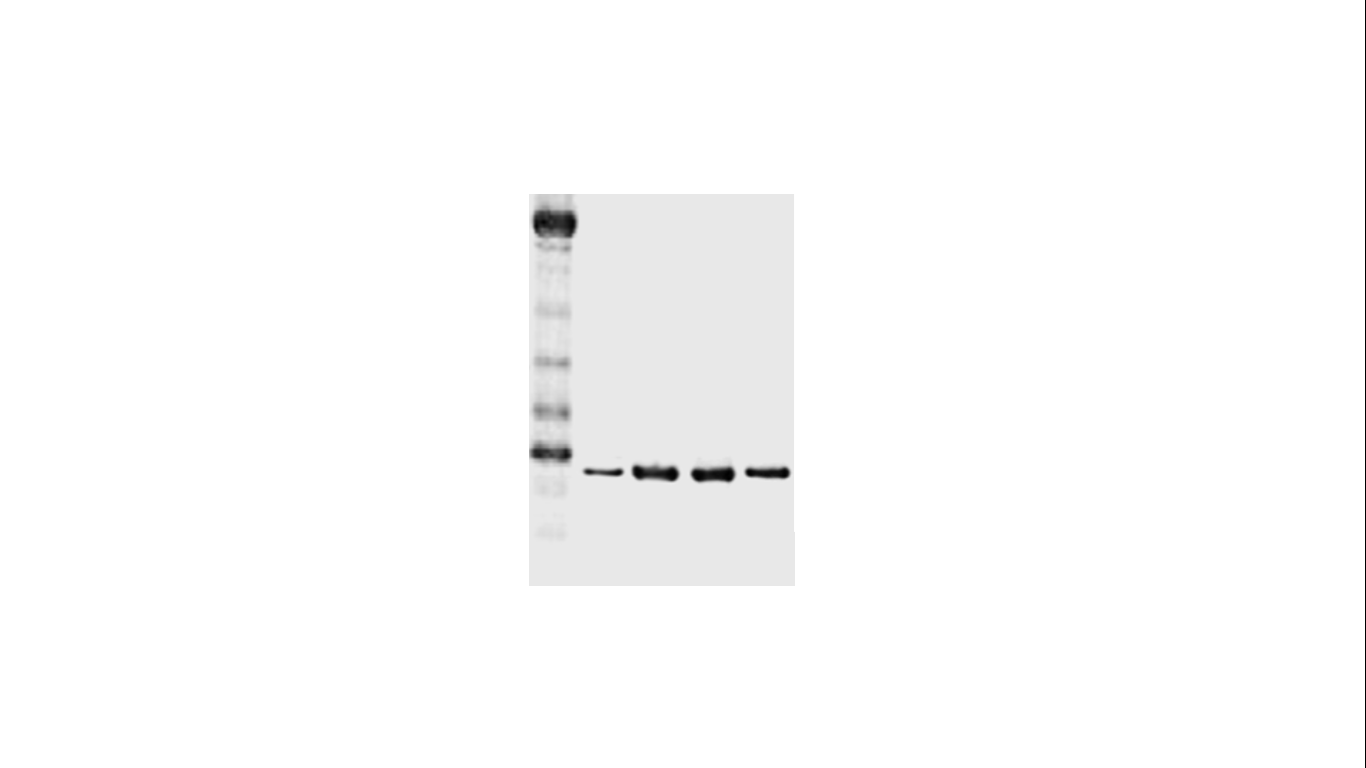
**Repeat 2**


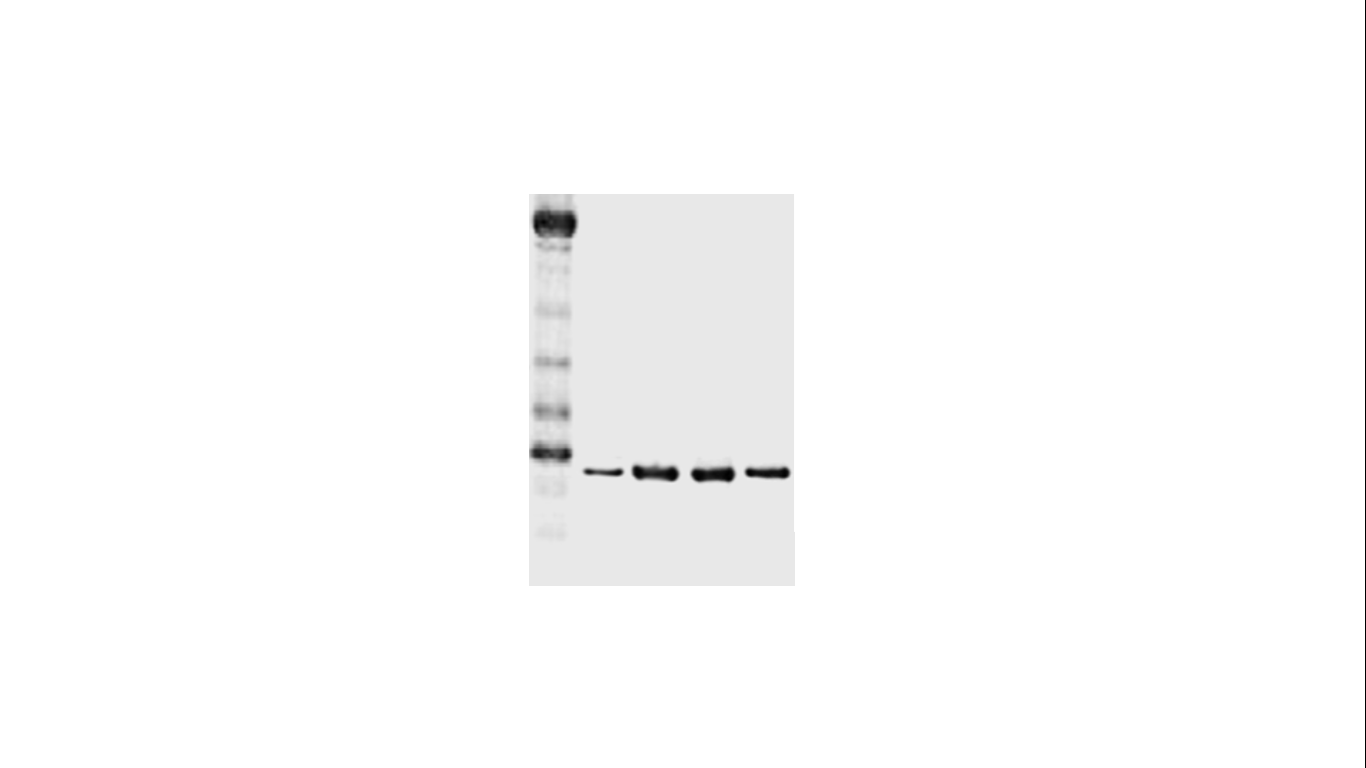
 ᵅ-SMA 42 KDa


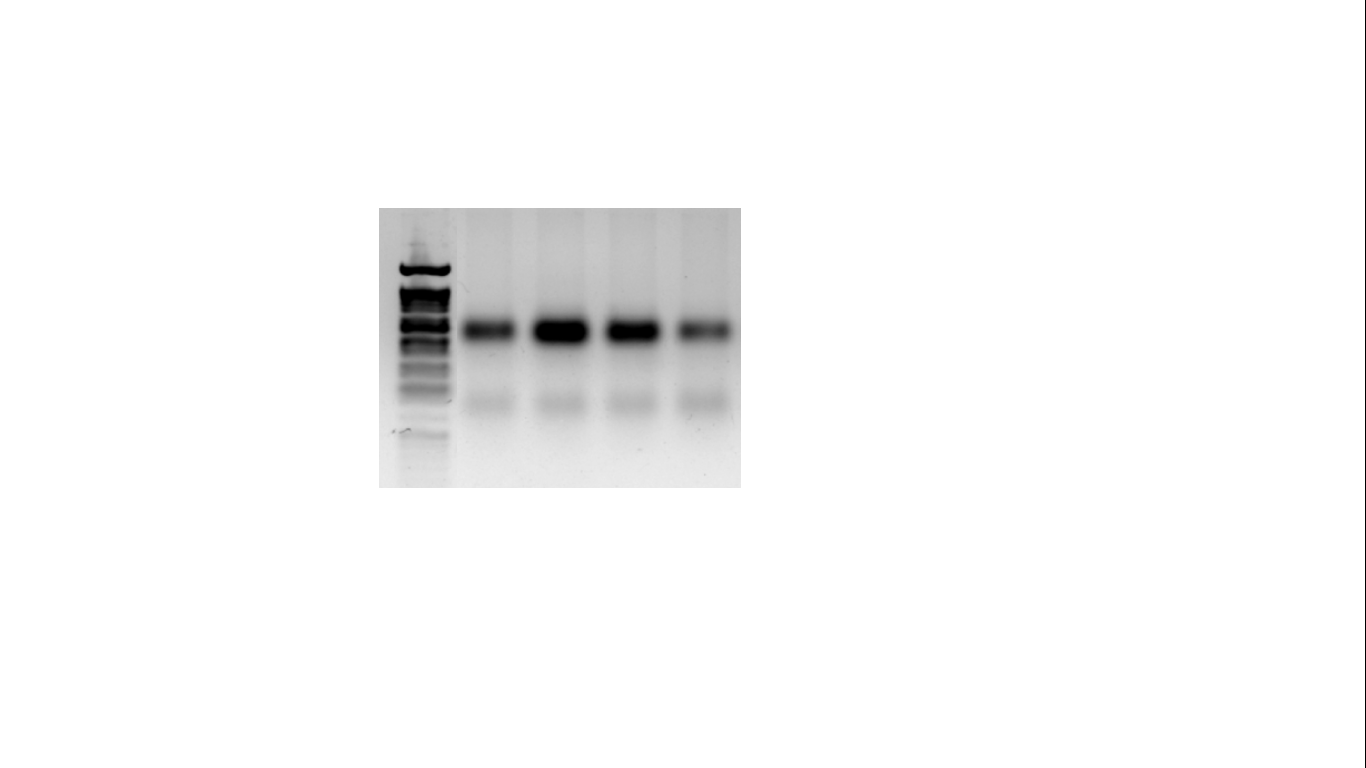
**Repeat 3**


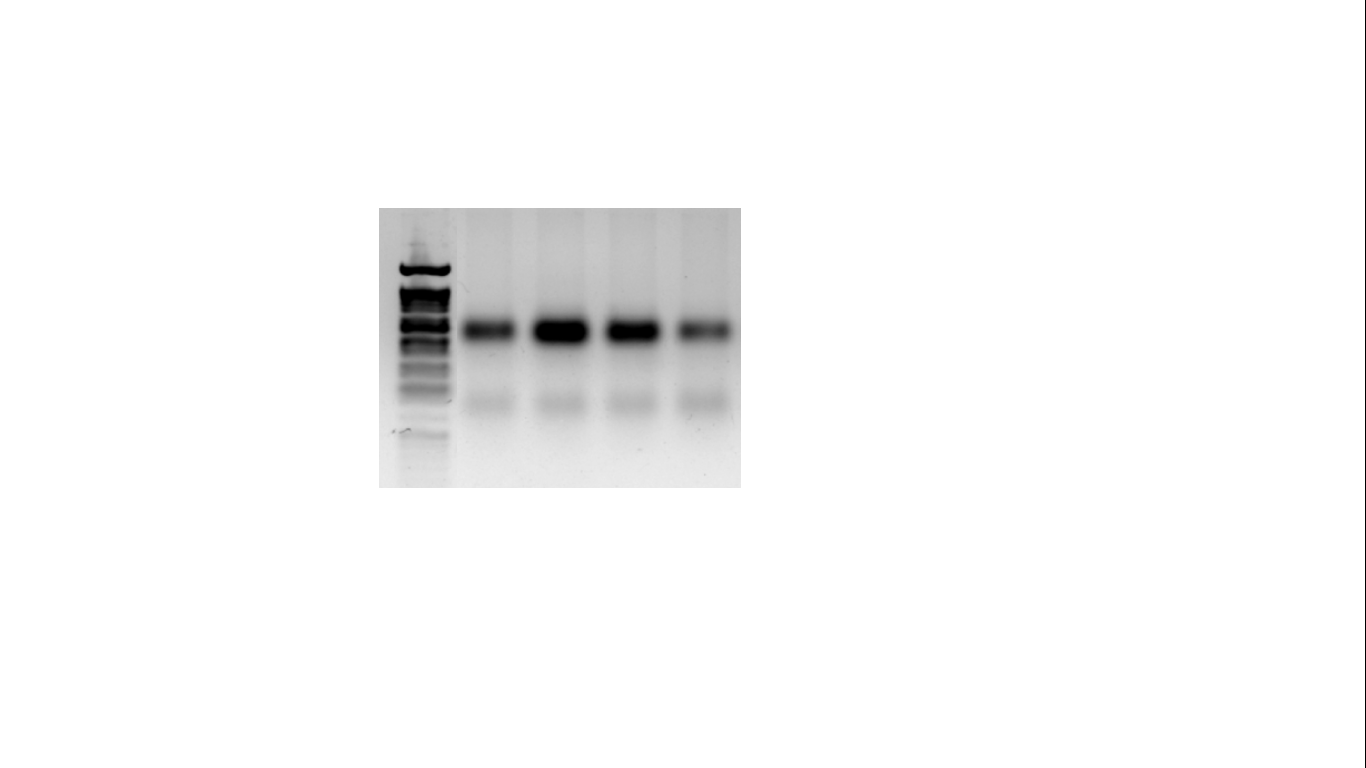
 ᵅ-SMA 42 KDa

**Collagen I**


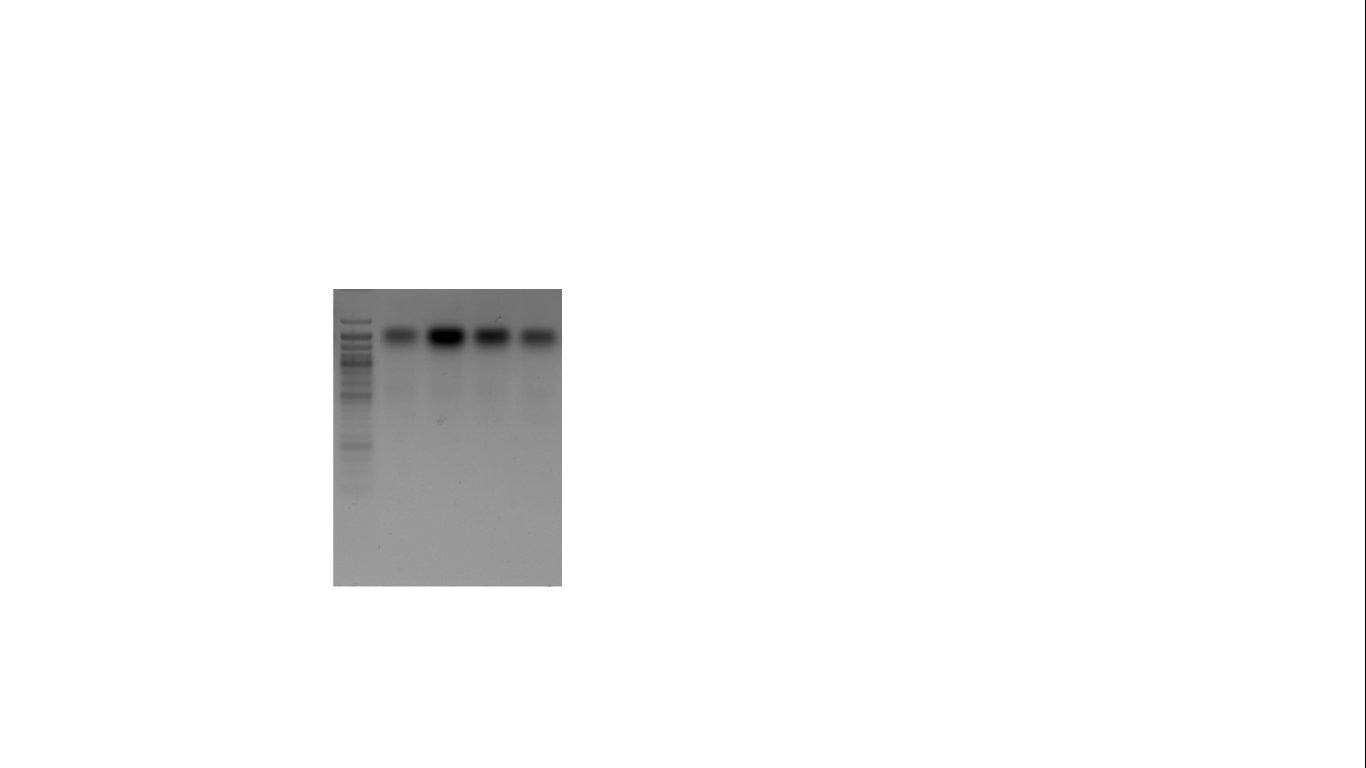
**Repeat 1**


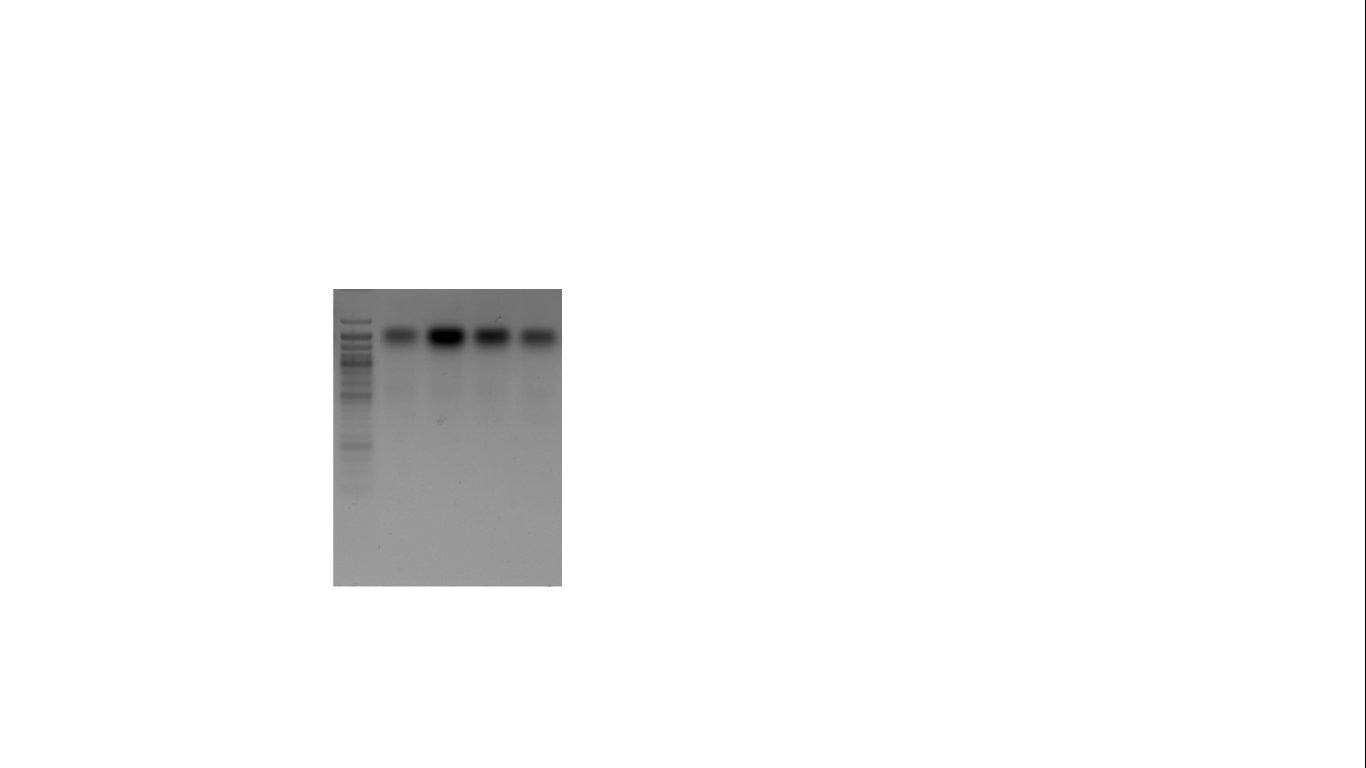


Collagen I 140 KDa

**Repeat 2**


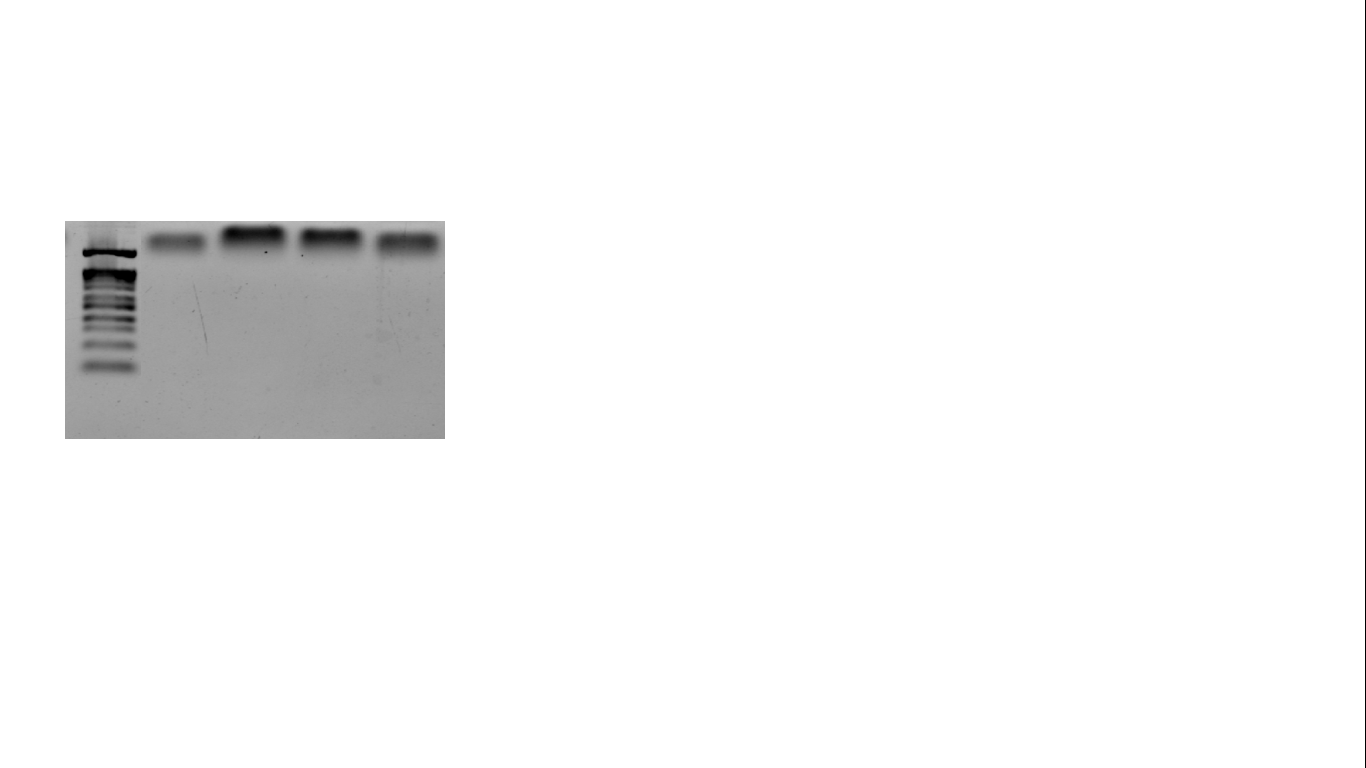


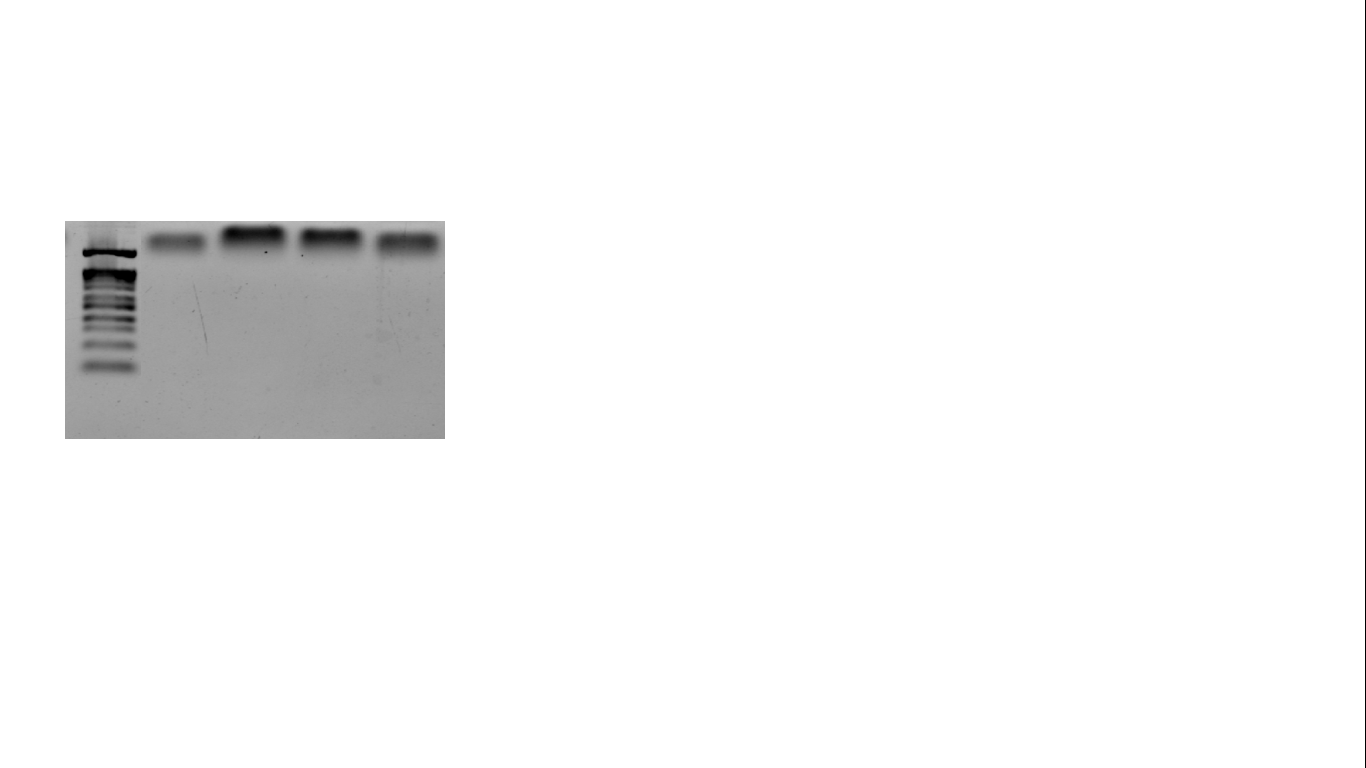


Collagen I 140 KDa

**Repeat 3**


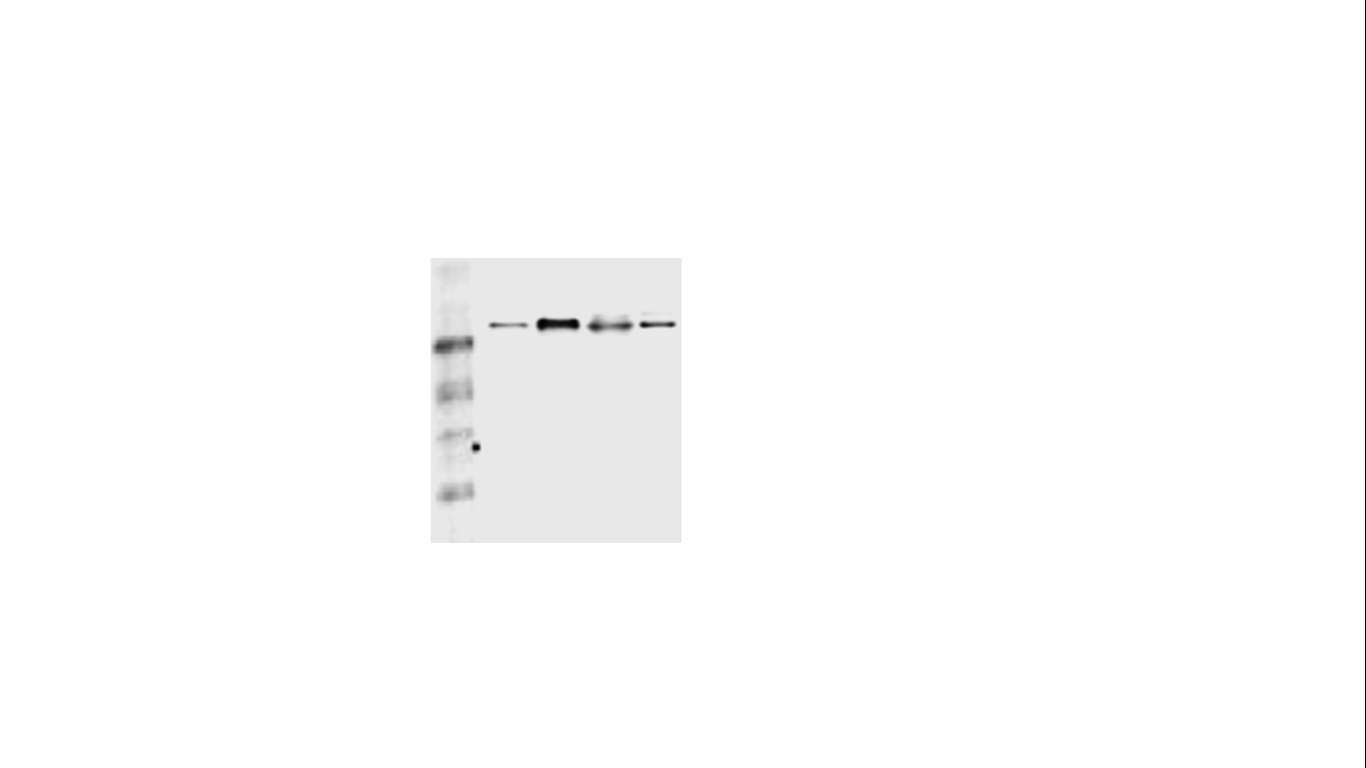


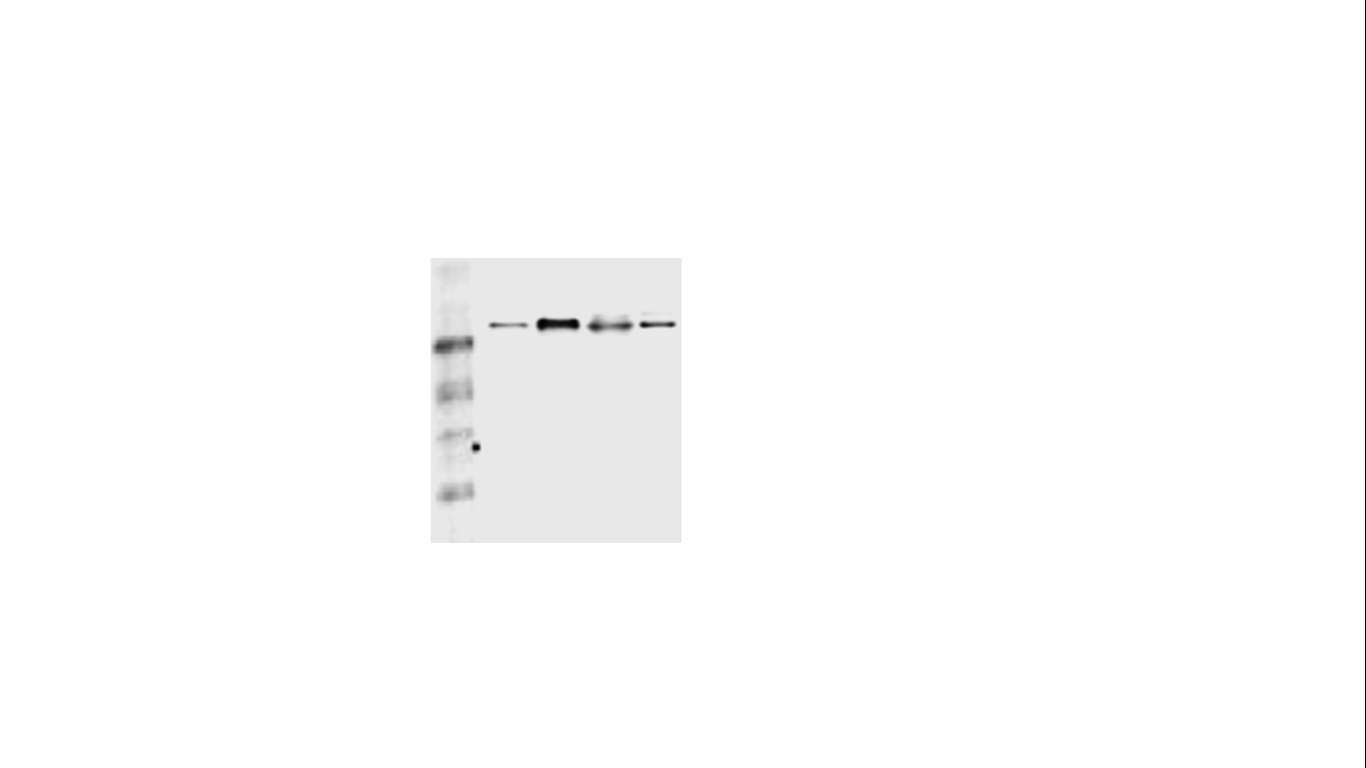


Collagen I 140 KDa

**Collagen** **III**

**Repeat 1**


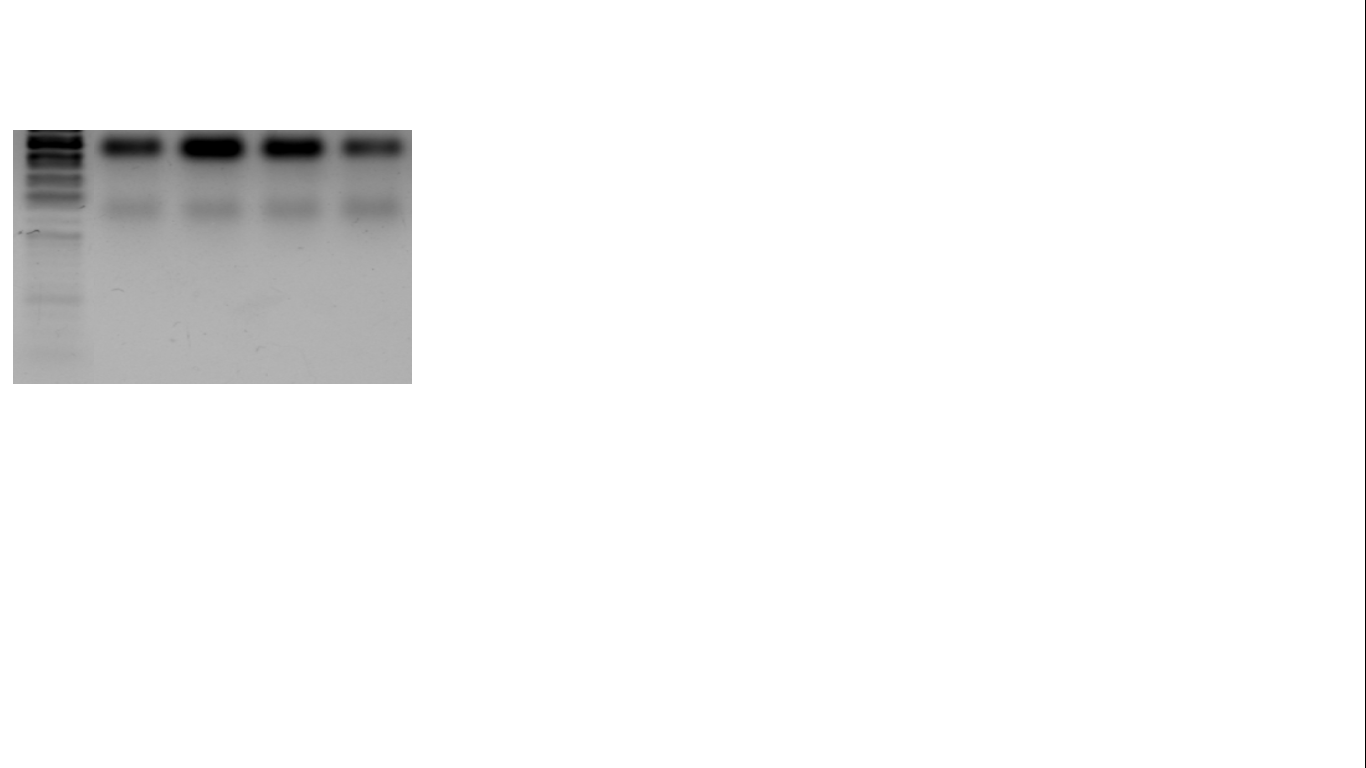


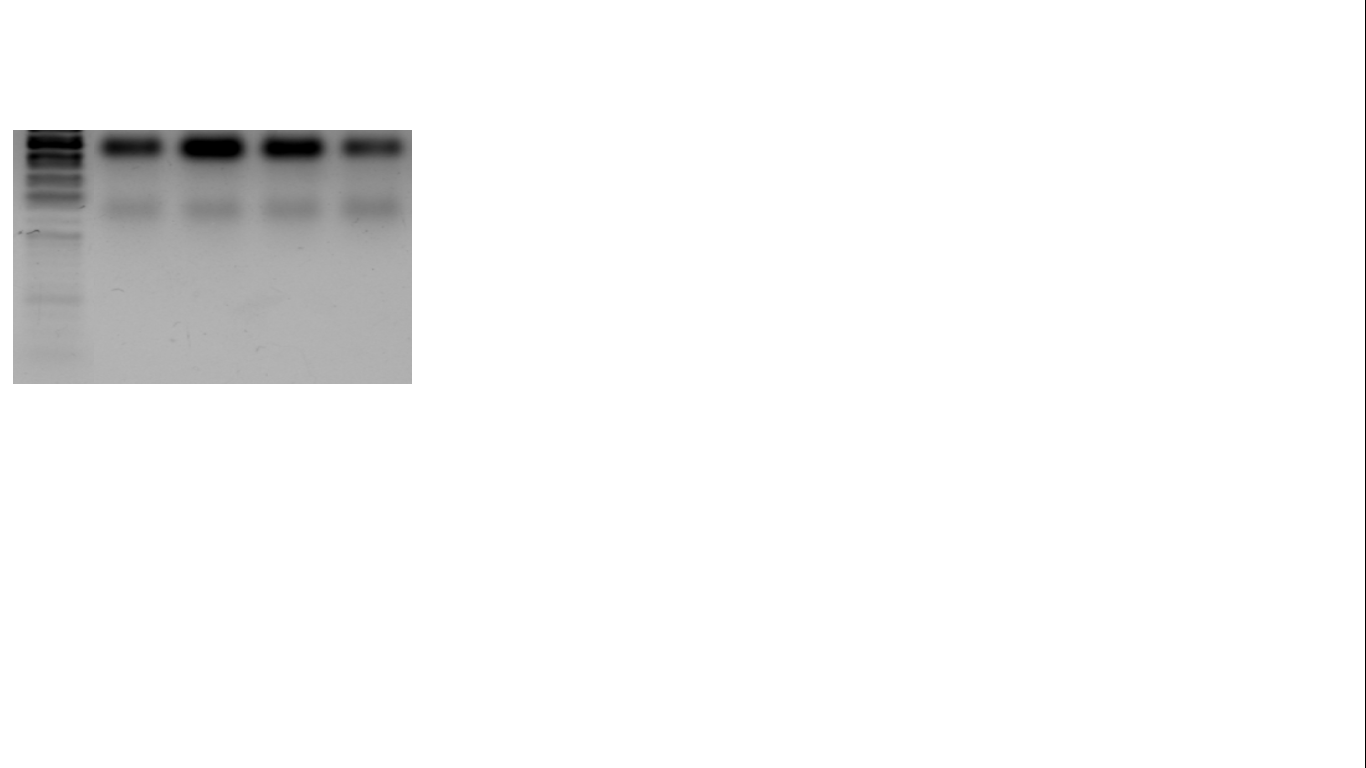


Collagen III 130 KDa


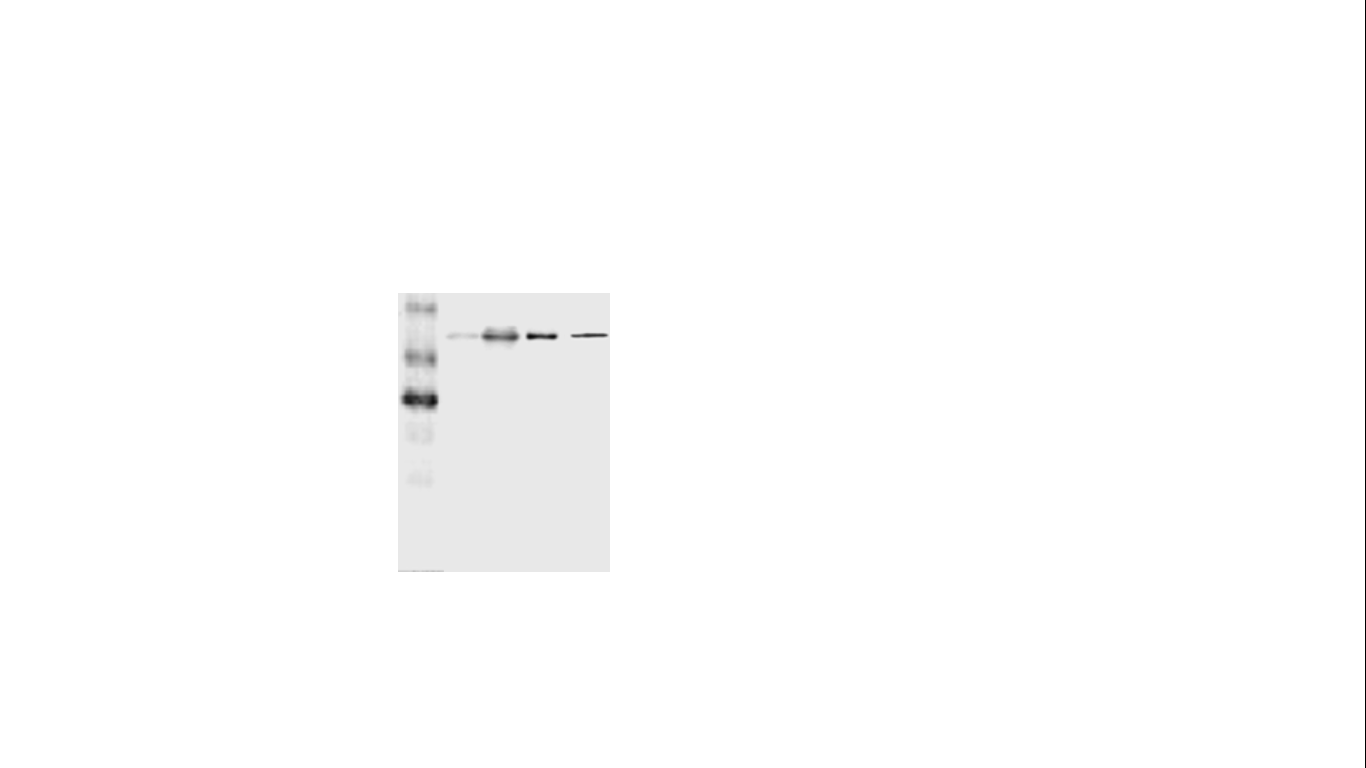
**Repeat 2**


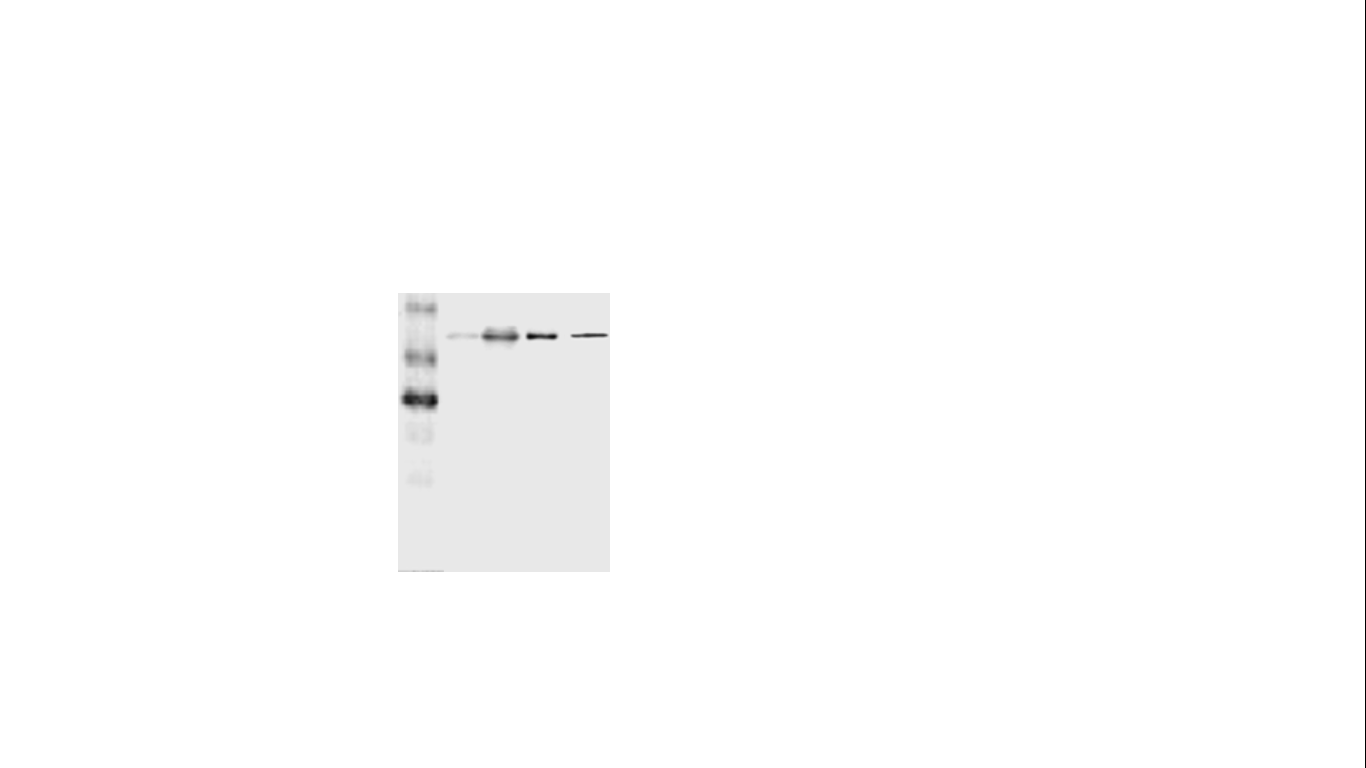


Collagen III 130 KDa

**Repeat 3**


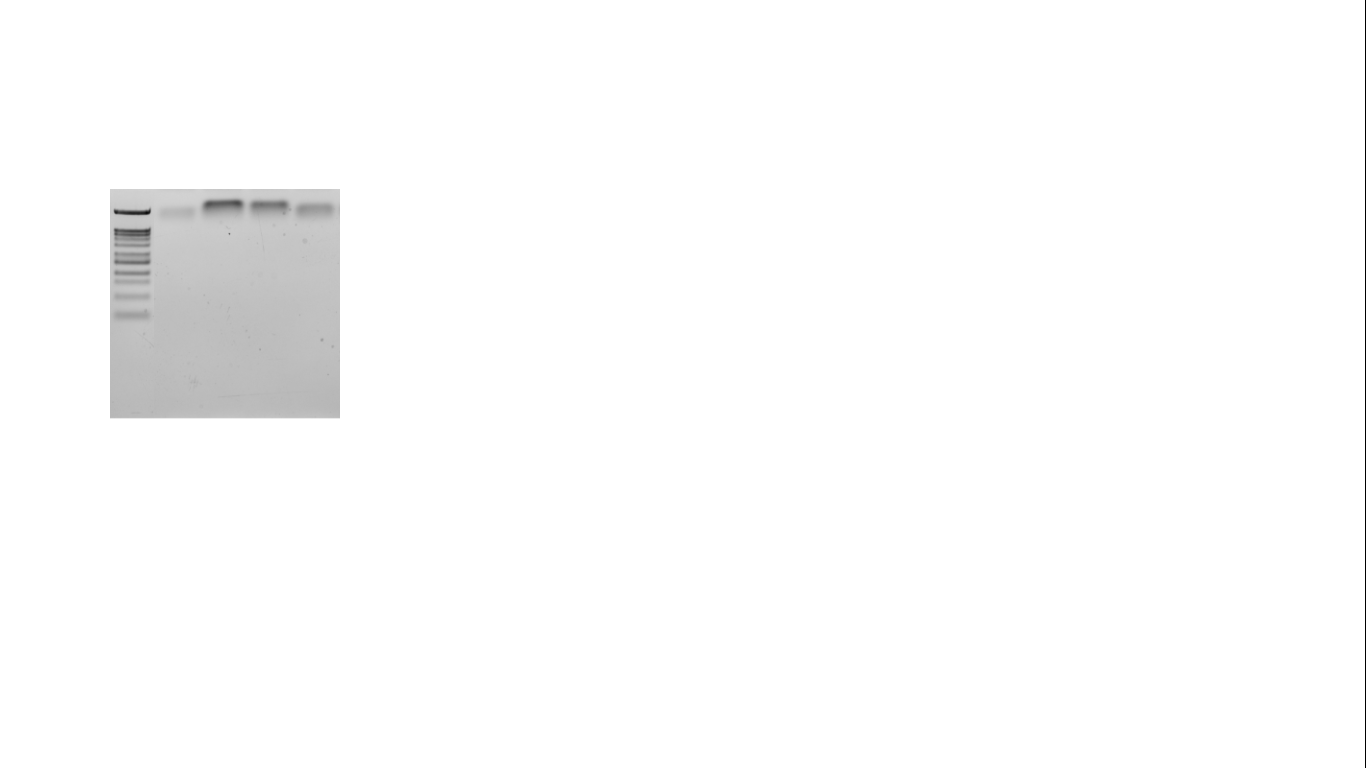


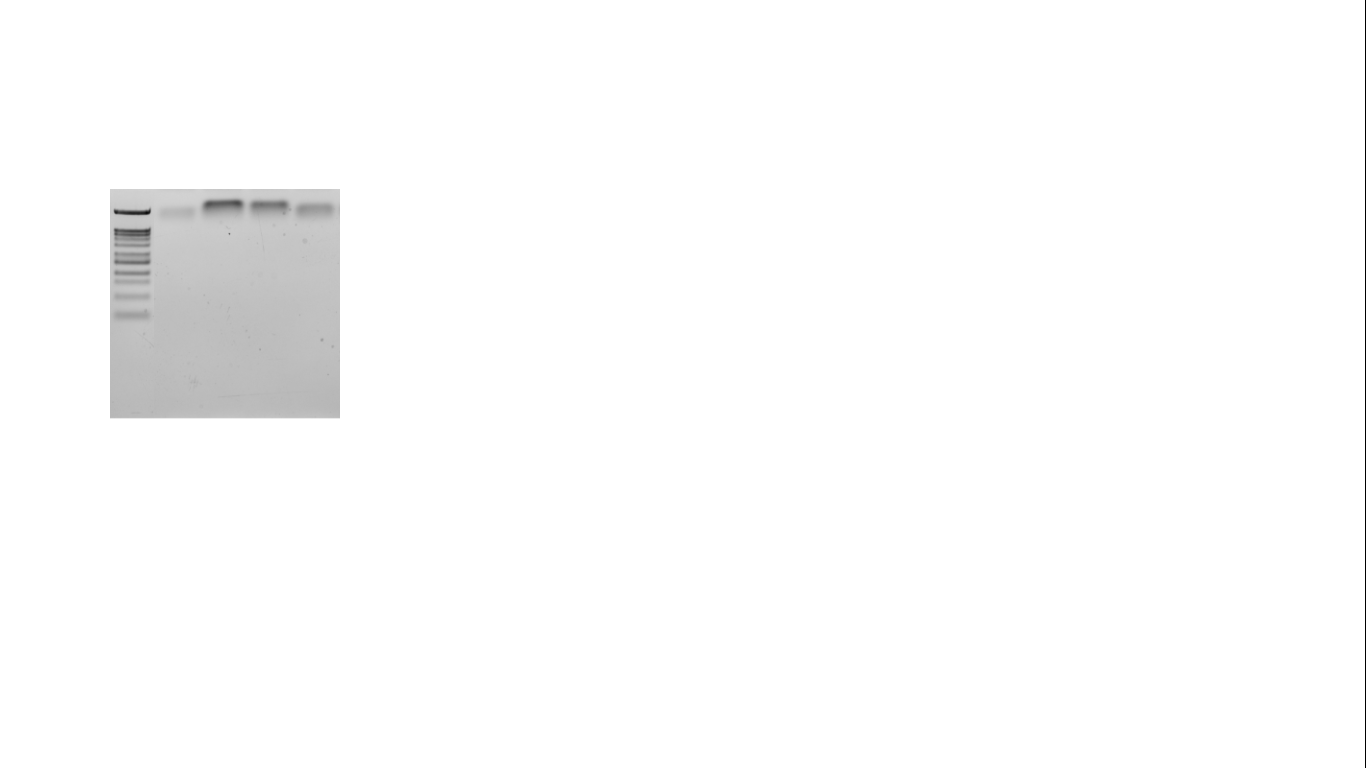
 Collagen III 130 KDa

**B-actin**


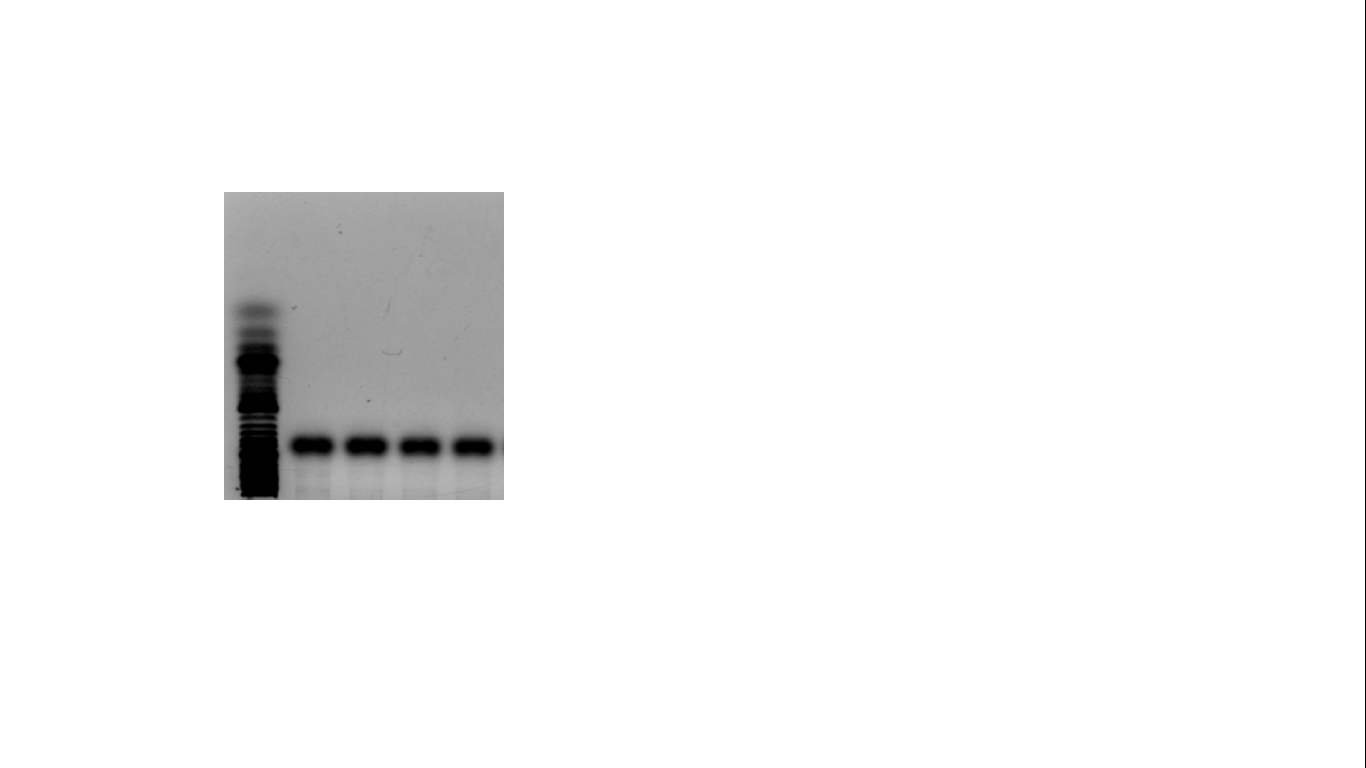
**Repeat 1**


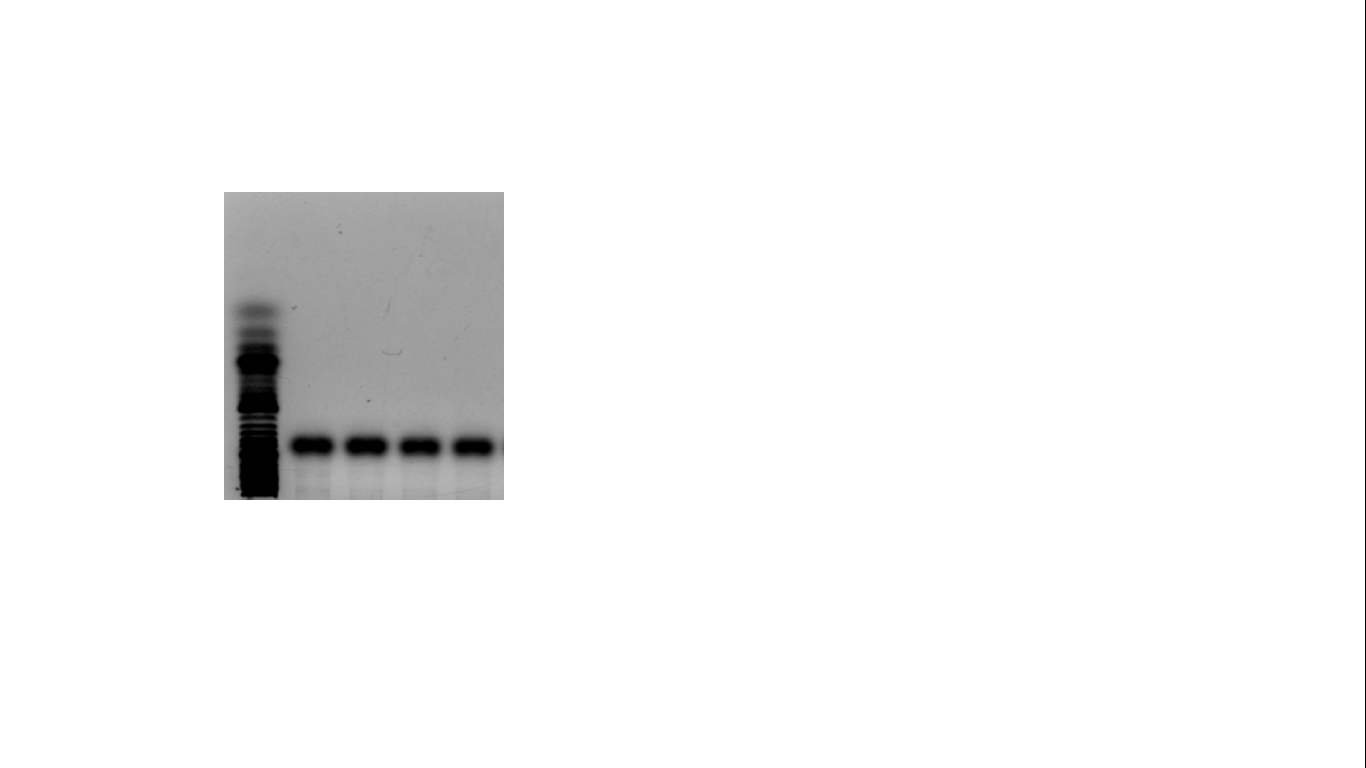


B-actin 43 KDa

**Repeat 2**


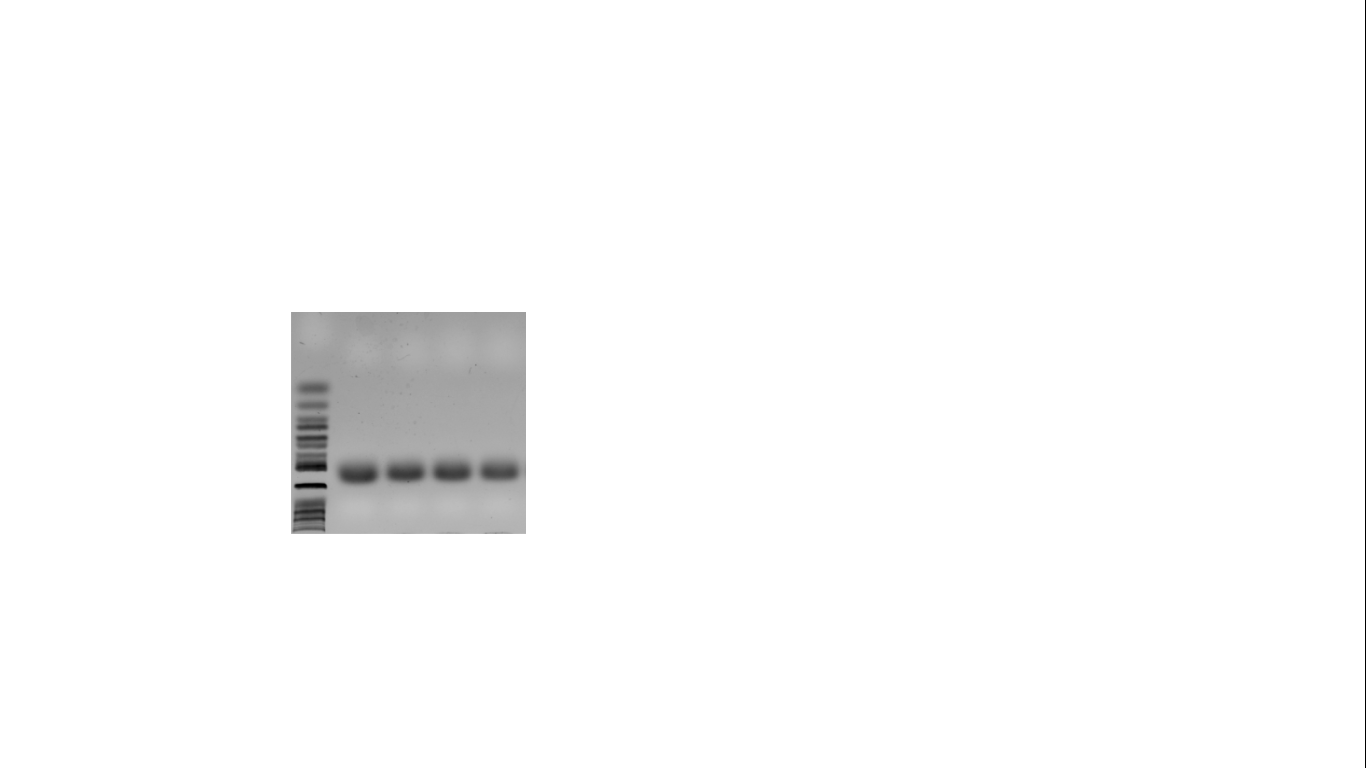


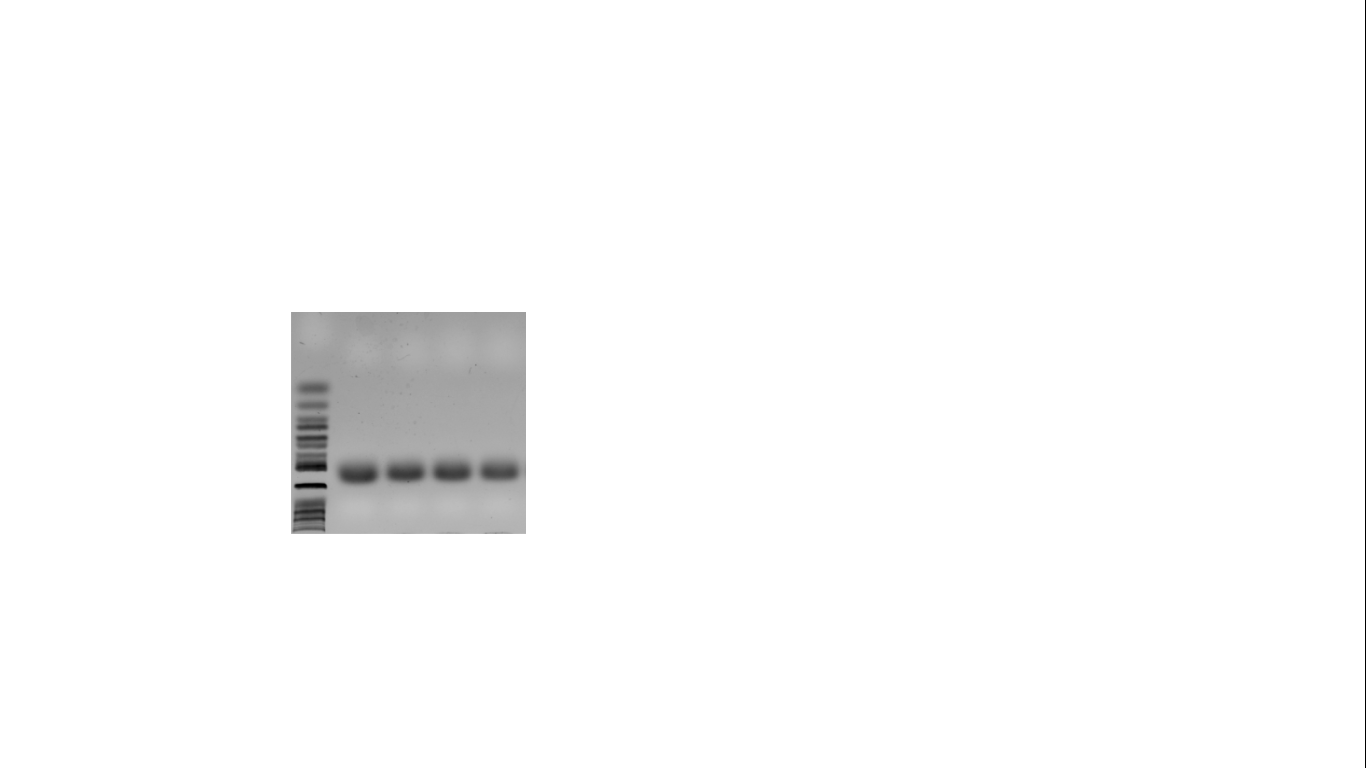


B-actin 43 KDa

**Repeat 3**


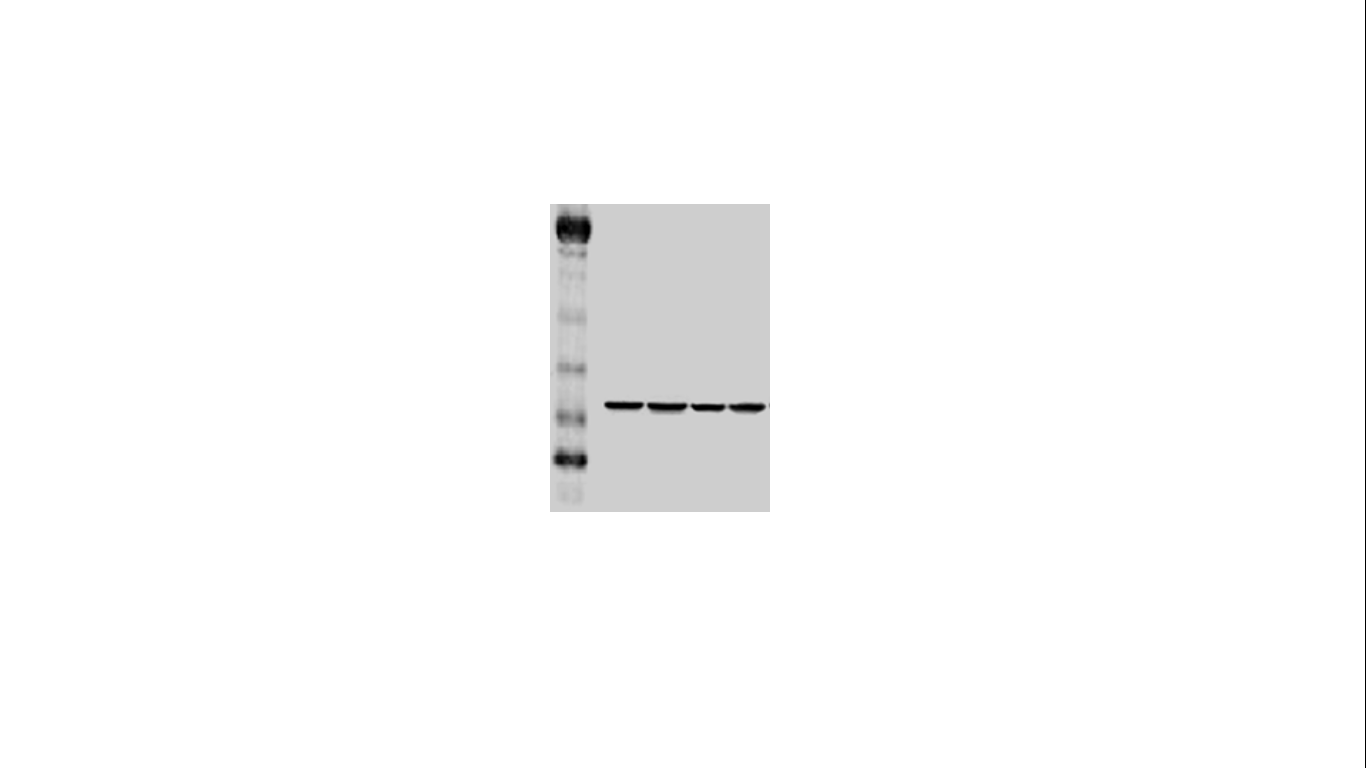


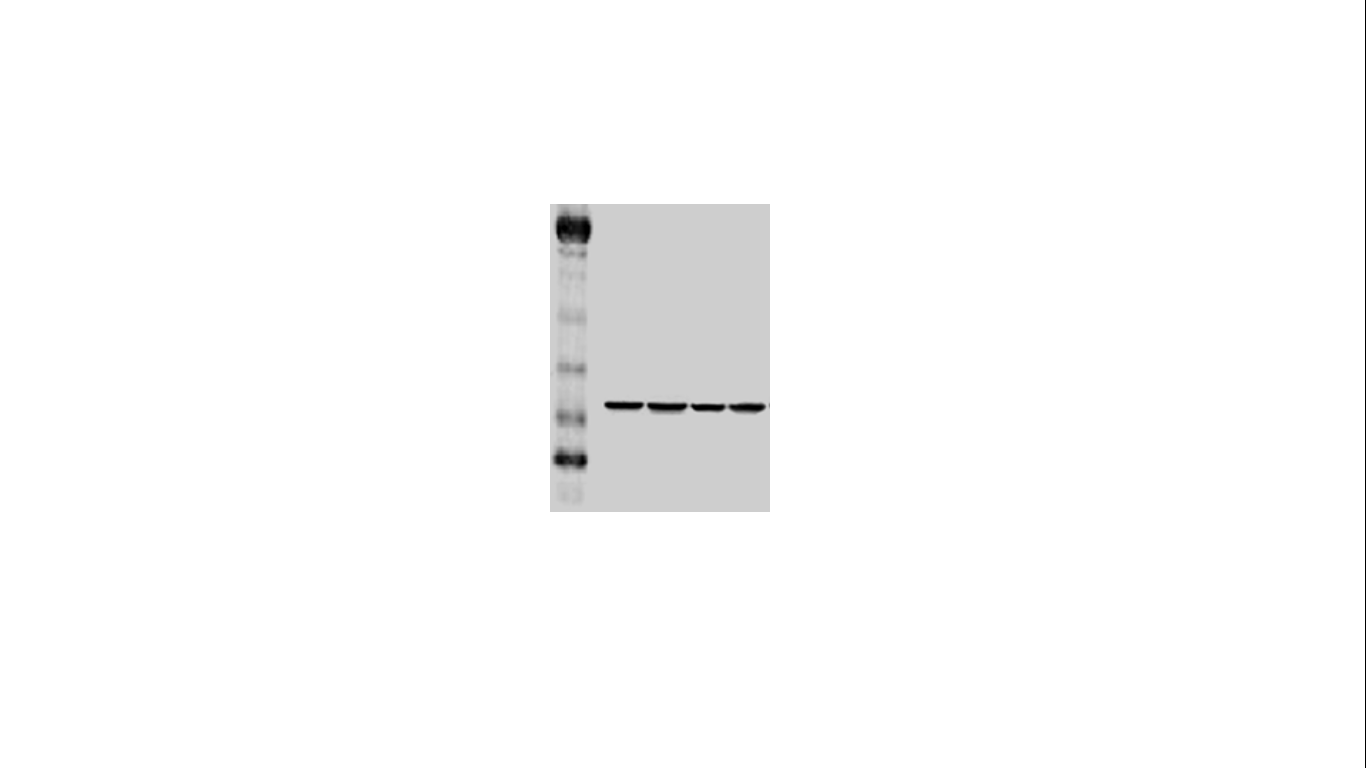
 B-actin 43 KDa
